# Glycovaccine Design: Optimization of Model and Antitubercular Carrier Glycosylation via Disuccinimidyl Homobifunctional Linker

**DOI:** 10.3390/pharmaceutics15051321

**Published:** 2023-04-23

**Authors:** Sara Tengattini, Davide Rubes, Massimo Serra, Luciano Piubelli, Loredano Pollegioni, Enrica Calleri, Teodora Bavaro, Gabriella Massolini, Marco Terreni, Caterina Temporini

**Affiliations:** 1 Department of Drug Sciences, University of Pavia, Via Taramelli 12, 27100 Pavia, Italy; davide.rubes01@universitadipavia.it (D.R.); massimo.serra@unipv.it (M.S.); enrica.calleri@unipv.it (E.C.); teodora.bavaro@unipv.it (T.B.); gabriella.massolini@unipv.it (G.M.); marco.terreni@unipv.it (M.T.); caterina.temporini@unipv.it (C.T.); 2 Department of Biotechnology and Life Sciences, University of Insubria, Via Dunant 3, 21100 Varese, Italy; luciano.piubelli@uninsubria.it (L.P.); loredano.pollegioni@uninsubria.it (L.P.)

**Keywords:** glycoconjugate vaccines, antitubercular vaccines, Ag85B antigen, chemical glycosylation, disuccinimidyl linkers, glycoconjugate characterization, glycosylation optimization

## Abstract

Conjugation via disuccinimidyl homobifunctional linkers is reported in the literature as a convenient approach for the synthesis of glycoconjugate vaccines. However, the high tendency for hydrolysis of disuccinimidyl linkers hampers their extensive purification, which unavoidably results in side-reactions and non-pure glycoconjugates. In this paper, conjugation of 3-aminopropyl saccharides via disuccinimidyl glutarate (DSG) was exploited for the synthesis of glycoconjugates. A model protein, ribonuclease A (RNase A), was first considered to set up the conjugation strategy with mono- to tri- mannose saccharides. Through a detailed characterization of synthetized glycoconjugates, purification protocols and conjugation conditions have been revised and optimized with a dual aim: ensure high sugar-loading and avoid the presence of side reaction products. An alternative purification approach based on hydrophilic interaction liquid chromatography (HILIC) allowed the formation of glutaric acid conjugates to be avoided, and a design of experiment (DoE) approach led to optimal glycan loading. Once its suitability was proven, the developed conjugation strategy was applied to the chemical glycosylation of two recombinant antigens, native Ag85B and its variant Ag85B-dm, that are candidate carriers for the development of a novel antitubercular vaccine. Pure glycoconjugates (≥99.5%) were obtained. Altogether, the results suggest that, with an adequate protocol, conjugation via disuccinimidyl linkers can be a valuable approach to produce high sugar-loaded and well-defined glycovaccines.

## 1. Introduction

The development of glycoconjugate vaccines requires the chemical conjugation of glycans to an immunogenic carrier protein. While most of the licensed conjugate vaccines are produced from polysaccharides extracted and purified from natural sources, current vaccine development strategies involve the use of well-defined minimal saccharide epitopes that are synthetized using chemical or enzymatic approaches [1,2,3]. Parallel to the selection of the sugar moieties, the choice of the conjugation strategy is also extremely important since it strongly influences the quality of the final glycoconjugates in terms of site-specificity, glycan loading, and purity/homogeneity of the final product.

The most common approach for the covalent coupling of oligosaccharide epitopes to carrier proteins entails introducing a spacer carrying reactive functional groups at their end terminal. Among the different options, the introduction of an amino group is one of the most convenient since it allows further derivatization with bifunctional or bivalent linkers to form active esters that are able to react with the ε-amino group of lysine residues on the carrier protein surface. The use of disuccinimidyl bivalent linkers (such as disuccinimidyl glutarate (DSG) or disuccinimidyl adipate (DSA)) that are able to react with amino-functionalized oligosaccharides to form reactive esters falls into that category [4], and several examples showing the application of this synthetic strategy to the development/production of glycovaccines can be found in the literature [5,6,7,8,9,10,11].

The presence of a linker with a variable number of C units allows desirable conjugation yields to be achieved in terms of the number of sugars covalently loaded on the carrier surface. However, the high tendency of disuccinimidyl-esters for hydrolysis may represent a significant drawback for this conjugation chemistry. This instability can hamper an extensive linker purification, leading to side-reactions between protein lysine residues and residual linkers, to form conjugates of the corresponding carboxylic acid [12]. Indeed, the reaction of amino-activated oligosaccharides with disuccinimidyl esters requires a high linker molar excess (>5 eq.) to avoid sugar-sugar dimer formation [13]. A purification step is therefore required to eliminate the linker excess that will preferentially react with the protein and form the corresponding acid in aqueous medium. Most of the protocols reported in the literature mention purification approaches based on liquid–liquid extraction and/or precipitation using different organic solvents, such as ethyl acetate or chloroform [5,6,7], or even water [13]. The effectiveness of these purification approaches has not been proven since, in most of these experimental works, glycan attachment to the carrier is monitored by SDS-PAGE and/or MALDI-TOF-MS analysis [6,7,9,11]. The use of these analytical methods only provides the average glycan loading, and no information regarding any side reactions or alterations occurring during conjugation, such as the conjugation with residual linker, can be obtained. Instead, the presence of contaminant conjugates, which alter protein mass, can affect the truthfulness of the determination. Another common approach for glycoconjugate characterization entails the use of phenol-sulfuric acid assay [5,7]. This method, exploiting sugar reactivity, is not affected by the presence of contaminant linker/acid conjugates; it still provides the average glycan loading, but there is no assurance of adequate purity.

Interestingly, when performed, a deep insight into the characterization of the glycoconjugates has revealed the presence of linker/acid conjugates [12]. Their presence not only reduces the glycan loading by occupying reactive sites, but it might strongly influence glycoconjugate properties and biological activity (even in an early development stage). 

Based on the above, it appears clear that the use of this conjugation chemistry to produce (potential) glycovaccines requires the development of most efficient purification protocols and a detailed analytical characterization of produced glycoconjugates. In this context, the aim of this work is to set up an experimental protocol to use a disuccinimidyl homobifunctional linker, namely DSG, to obtain glycoconjugates with satisfactory sugar loading and adequate purity that is suitable to be used as therapeutic agents. 

At first, a commercial model protein, ribonuclease A (RNase A, 13.7 kDa, 10 lysine residues) was considered to set up a suitable purification procedure and optimize conjugation conditions, as well as considering the reactivity of this conjugation chemistry. Accordingly, 3-aminopropyl small-sized (from mono- to tri-) mannose saccharides were ad hoc synthetized and used. Mannose-based saccharides were selected as a model since mannose is one of the main constituents of oligosaccharides used in glycoconjugate vaccines, both for mimicking bacterial surface polysaccharides (e.g., *Mycobacterium tuberculosis* arabinomannans) and/or targeting mannose receptor on antigen presenting cells [14]. Glycosylation outcomes have been discussed in comparison to an alternative conjugation chemistry since the same protein was used as model in a previous study; that study used an approach which required the activation of oligosaccharides as 2-iminomethoxyethyl (IME) thioglycosides were considered [15]. 

Once the suitability of this approach for glycoconjugate synthesis was demonstrated, it was applied to recombinant Ag85B (31.3 kDa, 8 lysine residues), one of the most potent *Mycobacterium tuberculosis* antigens [16,17], and to its mutated variant, Ag85B K30R-K282R (referred to as Ag85B-dm, 31.3 kDa, 6 lysine residues), developed by our research group [18].

Ag85B protein was selected and studied by us as a carrier in the development of a novel glycoconjugate vaccine with antitubercular activity [16]. In our rational design approach, the carrier protein was selected not only to be immunogenic, but also to exert a specific antitubercular activity synergic to the one showed by the glycan antigen. In a previous study, the predominant glycosylation of the two most reactive lysine residues of Ag85B, namely K30 and K282, was demonstrated to reduce intrinsic protein antigenicity [16,18]. To address the glycosylation separately from the protein epitope, K30 and K282 were thus substituted with arginine residues. The conservative substitution allowed protein antigenicity to be preserved and to avoid glycosylation at these positions [16,18,19]. The loss of the two most reactive sites had, however, the evident drawback of reducing the glycosylation efficiency in terms of the average number of incorporated saccharides [18]. Glycosylation via disuccinimidyl linker was herein also considered as a conjugation approach to glycosylate Ag85B-dm, with the aim of achieving adequate antigen loading and making it possible to exploit the potential of this optimized carrier in the future development of a novel antitubercular vaccine.

## 2. Materials and Methods

### 2.1. Materials and Instrumentations

Ethyl acetate (EtOAc), dimethylformamide (DMF), tetrahydrofuran (THF), dichloromethane (DCM), acetonitrile (can), di-succinimidyl glutarate (DSG), potassium phosphate, ribonuclease A (RNase A), chymotrypsin, dithiothreitol (DTT), and formic acid (FA) were purchased from Sigma-Aldrich (Milan, Italy). Trifluoroacetic acid (TFA) was purchased from PanReac AppliChem (Monza, Italy). Deionized water was obtained from a Milli-Q^®^ Integral purification system from Merck KGaA (Darmstadt, Germany).

Reactants and chemicals used for the synthesis of sugar derivatives were purchased from commercial sources (Sigma-Aldrich, Burlington, MA, USA (Merck KgaA group), Alfa Aesar Ward Hill, MA, USA (Thermo Fisher Scientific group) and used without further purification. Solvents were purified according to the guidelines described in *Purification of Laboratory Chemicals* [20] and were freshly distilled from the appropriate drying agent. THF was distilled from sodium/benzophenone ketyl, and DCM from CaH_2_. Reactions requiring anhydrous conditions were performed under N_2_. Compound purification was performed via flash chromatography using Silica Gel high-purity grade, pore size 60 Å 70–230 mesh, 63–200 μm (Sigma-Aldrich). Analytical thin layer chromatography (TLC) was performed on silica gel F254 precoated aluminium sheets (0.2 mm layer, Merck, Darmstadt, Germany), visualized by a 254 nm UV lamp, and stained with 5% H_2_SO_4_ in ethanol, followed by heating to 150 °C. Characterization of purified compounds was performed by NMR spectroscopy. NMR spectra were recorded on a Bruker Advance III 400 MHz spectrometer (Bruker Corporation, Billerica, MA, USA). High-resolution mass HRMS spectra were acquired using a X500B QTOF System (SCIEX, Framingham, MA 01701, USA) equipped with the Twin Sprayer ESI probe and coupled to an ExionLC™ system (SCIEX). Yields were calculated for compounds purified by flash chromatography and judged homogeneous by thin-layer chromatography, NMR, and mass spectrometry.

Chromatographic separations of 3-aminopropyl saccharides **7**, **11**, **18**, active esters **19**, **20**, **21** and glycoconjugates were performed on a Dionex UltiMate 3000 HPLC system (Thermo Scientific, San Jose, CA, USA) equipped with mobile-phase online degasser, ternary pump, autosampler, column thermostated compartment, and variable wavelength detector, and controlled by Chromeleon software (6.8 version). MS detection was achieved by using a linear ion trap mass spectrometer (LTQ) equipped with electrospray ion source (ESI) (Thermo Scientific, San Jose, CA, USA) and controlled by X-calibur software (2.0.7 version).

Antigenic proteins Ag85B wild-type (wt) and -dm were produced as recombinant proteins in *Escherichia coli* as previously described [17,18].

### 2.2. Synthesis of 3-Aminopropyl Saccharides

The chemoenzymatic synthesis of 3-aminopropyl monosaccharide and certain intermediate derivatives exploited in the preparation of previously undescribed 3-aminopropyl di/trisaccharides (*vide infra*) has been carried out by adapting known synthetic methods [14,21,22,23]. Detailed experimental procedures, complete characterization data, and copies of ^1^H- and ^13^C-NMR spectra for all new compounds are reported in the Supplementary Material.

### 2.3. Preparation and Purification of Activated Esters

#### 2.3.1. Functionalization of 3-Aminopropyl Saccharides

Functionalization of 3-aminopropyl saccharides was performed according to Wang et al. [7]. A mixture of **7**, **11**, or **18** (2 mg) and DSG (15 equiv) in DMF/phosphate buffer (100 mM, pH 8.0) (4:1 *v/v*, 0.3 mL) was gently stirred at room temperature. After 4 h, 1 µL of reaction mixture was diluted 1:100 with ACN and analyzed by HPLC-ESI-MS.

#### 2.3.2. HILIC-ESI-MS Monitoring of 3-Aminopropyl Saccharide Activation

HPLC-MS analysis of 3-aminopropyl saccharides and activated derivatives was performed using HILIC mode. The HILIC-ESI-MS method entailed the use of the Waters X-Bridge Amide column (3 × 150 mm; Waters Corporation, Milford, MA, USA) and a mobile phase composed of ACN + 0.1% FA (A) and water + 0.1% FA (B). Gradient elution was performed by linear increase from 15 to 45% B in 10 min. Temperature, flow rate, and injection volume were set to 50 °C, 0.35 mL/min, and 2 µL, respectively. The following MS parameters were applied: positive ion mode, scan range 150–800 *m/z* in full-scan mode, source voltage 4.6 kV, capillary voltage 49 V, sheath gas flow rate 45 (arbitrary units), auxiliary gas flow rate 20 (arbitrary units), capillary temperature 250 °C, tube lens voltage 250 V.

#### 2.3.3. Purification of Activated Esters by Washing with an Organic Solvent

Activated 3-aminopropyl saccharides were tentatively separated from excessive DSG according to Wang et al. [7]. The procedure entails washing and precipitation upon addition of 9 volumes of EtOAc to the reaction mixture, followed by further purification by washing with EtOAc 10 times and then drying.

#### 2.3.4. Purification of Activated Esters by HILIC Chromatography

Activated 3-aminopropyl saccharides were purified by HILIC chromatography with a Waters X-Bridge Amide column (3 × 150 mm; Waters Corporation, Milford, MA, USA) and a mobile phase composed of ACN (A) and water (B). Gradient elution was performed by linear increase from 10 to 35% B in 10 min. Temperature, flow rate, and injection volume were set to 35 °C, 0.35 mL/min, and 20–100 µL, respectively. UV absorbance was monitored at 214 nm. Fraction containing activated 3-aminopropyl saccharides was manually collected and dried under nitrogen flow.

### 2.4. Synthesis and Analytical Characterization of Glycoconjugates

#### 2.4.1. Conjugation Protocol (Optimized Conditions)

Proteins (RNase A, Ag85B wt and -dm) were dissolved in 100 mM phosphate buffer, pH 8 (concentration: 4 mg/mL) in presence of 3-aminopropyl esters **7**, **11**, **18** to a final glycoside/protein molar ratio of 100/1. The reaction mixtures were incubated for 16 h at 20 °C under continuous stirring. The conditions were set as reported after studying the influence of four different variables (molar ratio, protein concentration, buffer pH, and temperature) on glycosylation outcome by means of a Design of Experiments (DoE) approach (full factorial design, 2^k^, k = 4) To this aim, the open-source software Chemometric Agile Tool (CAT) was used (available freely on the site of the Italian Group of Chemometrics, http://www.gruppochemiometria.it/index.php/software (accessed on 2 March 2023).

#### 2.4.2. Intact Glycoconjugate HILIC-UV-ESI-MS Analysis

HPLC-MS analysis of intact glycoconjugates was performed by using HILIC mode, an AdvanceBio Glycan Mapping column (2.1 × 150 mm; 1.8 µm, Agilent Technologies, Santa Clara, CA, USA), and a mobile phase composed of ACN + 0.1% TFA (A) and water + 0.1% TFA (B). The elution gradient for RNase A glycoconjugates was set as follows: from 20% to 30% B in 1 min and from 30% to 45% B in 15 min. Elution gradient for Ag85B wt and -dm glycoconjugates was set as follows: from 20% to 25% B in 1 min and from 25% to 40% B in 15 min. Temperature, flow rate, and injection volume were set at 50 °C, 0.25 mL/min, and 2 µL, respectively. The following MS parameters were applied: positive ion mode, scan range 700–2000 *m/z* in full-scan mode, source voltage 4.5 kV, capillary voltage 8 V, sheath gas flow rate 25 (arbitrary units), auxiliary gas flow rate 5 (arbitrary units), capillary temperature 220 °C, and tube lens voltage 220 V.

#### 2.4.3. Chymotryptic Digestion of Glycoconjugate and Peptide Mapping Analysis by HILIC-UV-ESI-MS/MS

For the chymotryptic digestion (cleavage at carboxy-terminal position of methionine, tyrosine, phenylalanine, tryptophan, and leucine residues. Then, 25–100 μM protein solution in 50 mM ammonium bicarbonate (pH 8.5) was added with DTT in 100 mM ammonium bicarbonate (pH 8.5) to obtain a 10 mM final concentration. The solution was heated at 60 °C for 30 min to reduce the protein’s disulfide bonds, and then chymotrypsin was added to a final protein/enzyme ratio of 50:1 (*w/w*). Then the solution was incubated for 3 h at 37 °C under continuous stirring. The reaction was stopped by adding 2.5% (*v/v*) TFA.

Glycopeptide mapping was performed by HILIC-UV-ESI-MS^3^. The column was the AdvanceBio Glycan Mapping column (2.1 × 150 mm; 1.8 µm, Agilent Technologies, Santa Clara, CA, USA) and the mobile phase was composed of ACN + 0.05% TFA (A) and water + 0.05% TFA (B). Elution gradient was set as following: isocratic at 5% B for 0.5 min; linear gradient from 5 to 40% B in 45 min. Temperature, flow rate and injection volume were set at 50 °C, 0.25 mL/min and 15 µL, respectively. UV absorbance was collected at 215 nm. MS detection required the following instrumental conditions: positive ion mode, source voltage 4.5 kV, capillary voltage 31 V, sheath gas flow rate 40 (arbitrary units), auxiliary gas flow rate 10 (arbitrary units), capillary temperature 250 °C, and tube lens voltage 95 V. Full scan mass range was set up from 300 to 2000 Da. MS^2^ and MS^3^ spectra were obtained by collision induced dissociation (CID) with normalized collision energy of 35.0. Data processing was performed using Bioworks Browser (Thermo Fisher Scientific (Waltham, MA, USA), revision 3.1).

## 3. Results and Discussion

### 3.1. Synthesis of 3-Aminopropyl Saccharides

**Scheme 1:**
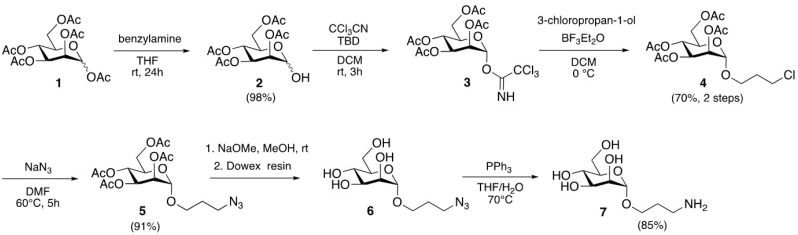
Preparation of monosaccharide **7**.

The monosaccharide **7** (Scheme 1), endowed with an aminopropyl chain at anomeric position, was prepared by exploiting a slightly different synthetic strategy than those previously described by Cramer [21] and Zhang [22]. Commercial peracetylated mannose **1** was selectively deprotected at the anomeric position with benzylamine to obtain **2** in 98% yield, and then converted to trichloroacetimidate-activated sugar **3** by treatment with trichloroacetonitrile in the presence of polystyrene supported 1,5,7-triazabicyclo[4.4.0]dec-5-ene (TBD) as a base. The installation of the chlorinated side chain was performed through reaction of crude **3** with 3-chloropropan-1-ol, which afforded **4** in 70% overall yield. The subsequent displacement of the chlorine atom of **4** with sodium azide in N,N-dimethylformamide (DMF) gave the corresponding azidopropyl derivative **5** in 91% yield. The completion of the synthesis included deprotection of peracetylated **5** with sodium methanolate to gain **6**, and reduction of the azide moiety by means of a Staudinger reaction with triphenylphosphine to obtain **7** (85% yield over two steps). It is worth noting that our synthetic sequence allowed the preparation of the desired amino sugar **7** in 6 steps and provided 53% total yield, which is higher than the 43% and 16% total yields reported by Cramer [21] and Zhang [22], respectively. 

**Scheme 2:**
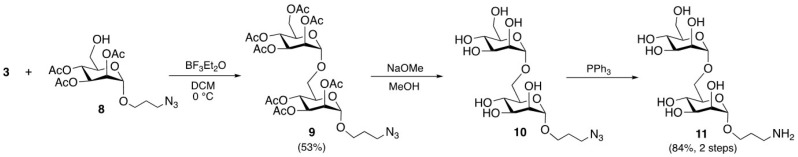
Preparation of disaccharide **11**.

The disaccharide analogue **11** (Scheme 2) was synthesized starting from the trichloroacetimidate-activated building block **3** and the 6-hydroxy azidopropyl derivative **8**, easily obtainable by submitting the previously described compound **5** to a regioselective enzymatic cleavage of the acetyl group at C6 [14]. In the presence of boron trifluoride diethyl etherate, the glycosidic donor **3** reacted with acceptor **8** to give the peracetylated disaccharide **9** in 53% yield. The required 1-aminopropyl derivative **11** was obtained, as stated for **7**, through removal of the acetyl protecting groups with sodium methanolate and Staudinger reduction of the azido group. 

**Scheme 3:**
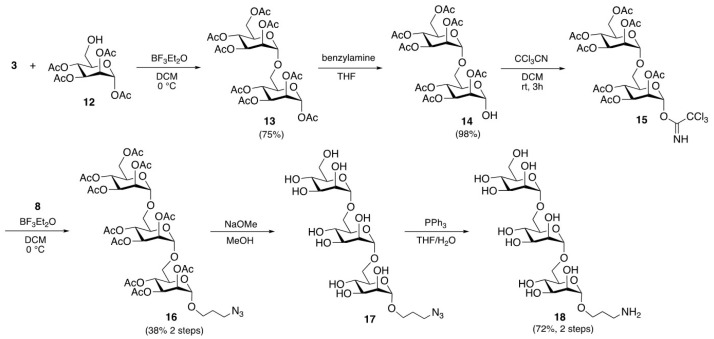
Preparation of trisaccharide **18**.

The synthesis of trisaccharide **18** (Scheme 3) was started with the condensation between trichloroacetimidate-activated mannosyl derivative **3** and 6-OH glycosidic acceptor **12** [14]. The disaccharide **13**, isolated in 75% yield, was then converted into the trichloroacetimidate **15**. The formation of a glycosidic bond between this last derivative and the azidopropyl building block **8** allowed the isolation of the fully protected trisaccharide **16** in 38% yield over two synthetic steps. Even in this case, the installation of the amine moiety onto the anomeric side chain of **18** was accomplished via transesterification of the acetyl groups of **16** with sodium methanolate followed by reduction of the azide moiety (72% yield over two steps). 

### 3.2. General Procedure for Glycoconjugate Synthesis

**Scheme 4:**
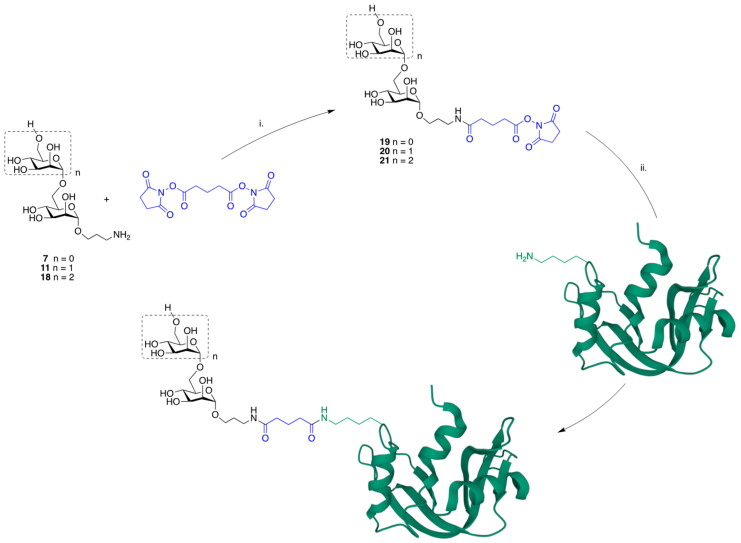
(i) DSG (15 equiv.) in DMF/100 mM phosphate buffer, pH 8.0 (4:1 *v/v*), room temperature, 4 h. (ii) 100 mM phosphate buffer, pH 8, 20 °C, 16 h. Protein graphical representation depicts model protein RNase A and was taken from the Protein data Bank (PDB DOI: 10.2210/pdb2E3W/pdb).

The preparation of the desired glycoconjugates involved two different steps. First, the aminopropyl saccharides **7**, **11,** and **18** were modified through the formation of an amide bond between their amino group and one of the terminals of the homobifunctional DSG linker. Then, the obtained active esters **19**–**21**, purified from linker excess, were reacted with the ε-amino group of lysine residues on the protein surface (Scheme 4).

### 3.3. Functionalization of 3-Aminopropyl Saccharides **7**, **11**, **18** via DSG Linker

The synthetized 3-aminopropyl saccharides were functionalized through reaction with DSG linker. Reaction was performed according to the procedure reported by Wang et al. [7]. HILIC-MS monitoring of reaction mixtures showed the complete disappearance of 3-aminopropyl saccharide **7**, **11,** and **18** after 4 h, to give the respective active esters. 

### 3.4. Active Ester Purification 

#### 3.4.1. Purification by Precipitation with an Organic Solvent

**Scheme 5:**
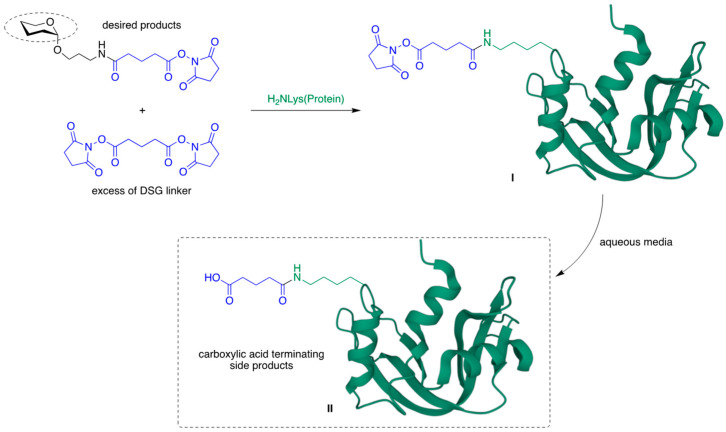
Side products arising from inadequate linker removal.

After derivatization with the DSG linker, the synthesized esters **19**–**21** require careful purification to quantitatively remove the excess linker. Indeed, if present, the residual linker can react with the carrier protein, giving acylated side products of the type **I**, which in aqueous media can be hydrolyzed to give the corresponding carboxylic acid terminating side products **II** (Scheme 5).

Most of the literature references reports purification protocols using multiple washes with EtOAc, theoretically resulting in active ester precipitation and quantitative removal of residual DSG [7]. This procedure was applied to **19** and **20**. A 9-volume wash with EtOAc was performed, and the resulting precipitate was dried, solubilized in DMF, and injected in RP-HPLC-UV-ESI-MS. The procedure was then repeated by washing the precipitate with and additional 10-volume of EtOAc. Values of chromatographic peak areas allowed for the estimation of percentual decreases of **19** and **20**, as well as DSG, during purification. For **19,** we observed a significant loss of product (around 50%). It is likely that the presence of only one sugar residue does not make the resulting ester sufficiently hydrophilic to avoid its solubilization in EtOAc. This behavior is thus strictly related to sugar chain dimension, as confirmed by the fact that the same procedure resulted in a 15% lower loss (around 35%) in the case of **20**. Interestingly, residual linker was not quantitively removed by the applied procedure: DSG peak was still detectable even after the 19-volume washes in both samples. Data are summarized in Table S1.

Despite this evidence, a tentative conjugation of **20** with the carrier model protein, RNase A, has been performed in non-optimized conditions (1 mg/mL protein concentration in 100 mM phosphate buffer, pH 8; molar ratio ester/protein, 30:1 (theoretical); 37 °C; 96 h). RNase A is a small protein (13.7 kDa) presenting 10 lysine residues as potential conjugation sites.

**Figure 1:**
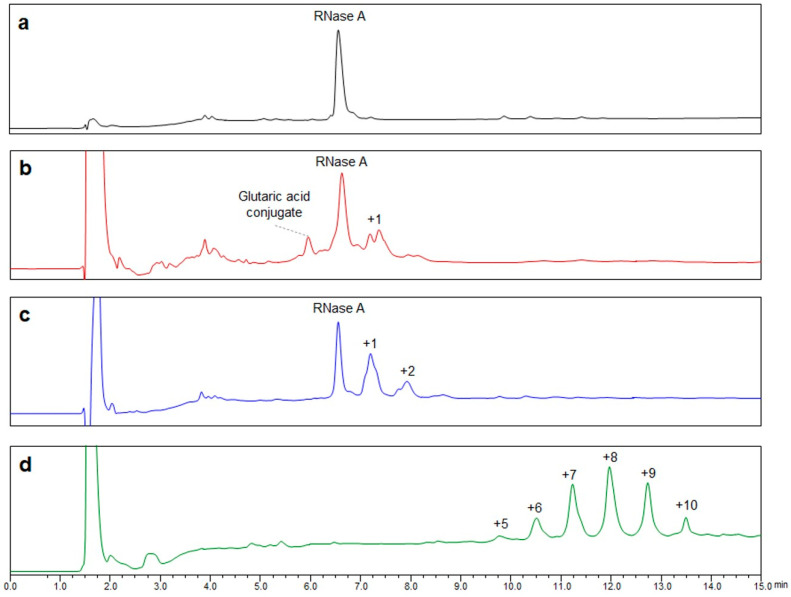
HILIC-UV profile of (**a**) RNase A; (**b**) RNase A conjugated with **20** purified by precipitation with EtOAc, conjugation performed in non-optimized conditions; (**c**) RNase A conjugated with **20** purified by HILIC, conjugation performed in non-optimized conditions; (**d**) RNase A conjugated with **20** purified by HILIC, conjugation performed in optimized conditions. Detection was performed at 214 nm. Numbers indicate the incorporated Man_2_ units.

Since the resulting product was expected to be a complex mixture of protein, glycoforms (with different number of incorporated saccharides and different glycosylation sites) and side products (from the conjugation with residual presence of DSG), a highly selective chromatographic HILIC method was ad hoc developed to resolve the different species and simplify MS detection. Figure 1a shows the elution profile obtained for RNase A with the optimized HILIC method. The chromatogram of the product obtained from the conjugation of **20** and RNase A is reported in Figure 1b. In the chromatographic trace, the unmodified protein is present as main species, followed, at higher retention times, by the peak corresponding to the mono-glycosylated species. The peak splitting can be ascribed to different glycosylation sites, since they can differently impact on glycoprotein interaction with HILIC stationary phase, thus resulting in a partial separation [24]. The peak eluting at lower retention time, well resolved from RNase A, showed a deconvoluted mass of 13,795 Da, with a mass shift of +114 Da from the unmodified protein. This species resulted from the conjugation of RNase A to residual linker, forming a glutaric acid conjugate. While the unsatisfactory glycosylation yield can be ascribed to the lack of correspondence between the theoretical and the actual ester/protein molar ratio, due to the unsuitability of this purification protocol for low-size saccharides, the results also demonstrated that this procedure does not guarantee the quantitative removal of unreacted linker, yielding an unacceptable glycoconjugate purity degree.

#### 3.4.2. Development of an Alternative Purification Protocol

The results reported in the previous section clearly assessed the need to develop an alternative purification protocol for DSG-activated aminopropyl saccharides.

First, solid phase extraction (SPE) was considered by investigating different solid phase materials. Normal phase (NP)-SPE was the first choice due to the use of non-protic solvents, followed by porous graphitized carbon material (PGC)-SPE, and reverse phase (RP)-SPE. As the solid phase strongly retained both the linker and the activated esters, no separation was achieved with PGC-SPE; NP-SPE and RP-NP showed a different (and opposite) selectivity for active esters and the DSG, allowing them to be successfully collected in different fractions. However, glycosylation of RNase A with active esters purified by SPE still resulted in unsatisfactory yields and, notably, in contamination by glutaric acid conjugates (data not shown). The HPLC-MS analysis of the fractions containing the active esters pointed out the presence of the mono-hydrolyzed linker as contaminant. Since the reaction between 3-aminopropyl saccharides and DSG takes place in the presence of 20% (*v/v*) phosphate buffer (see Section 2.3.1), a collateral reaction leads to the partial (or total) hydrolysis of DSG. The resulting mono- or di- carboxylic acids, due to the increased hydrophilicity, can elute with the saccharide active esters in both NP- and RP-SPE. Their presence in the glycosylation mixtures significantly affects the outcome of the reaction. The dicarboxylic acid (glutaric acid) is a non-reactive species, but its presence alters the reaction pH, while the monocarboxylic acid still possesses an active ester group that can react with lysine residues and form side-products.

SPE experiments pointed out that a more selective purification method of active esters was needed, addressing our investigation to the chromatographic step: HILIC is the chromatographic mode of choice for the analysis/separation of glycans. Moreover, mimicking NP conditions, HILIC requires a mobile phase that is mainly composed of organic solvent (ACN) with a low percentage of water (5–20%). For these reasons, it was considered as a preparative tool for the purification of activated esters. A carbonamide column from Waters, X-Bridge Amide, was selected and exploited for the purification of active ester reaction mixtures. A representative HILIC-UV chromatogram, obtained by the injection of 1 µL of reaction mixture containing **21**, is reported in Figure S2. While the main peak corresponds to active ester, other side products were detected and separated; their presence may affect the glycosylation outcome. Moreover, the separation method also allowed the percentage of product hydrolysis to be estimated during purification, which is the most common degradation reaction occurring in esters. In the case of **21,** the degradation was estimated by the UV area as 2.6% (peak at rt 12.157 min in Figure S2). The desired amount of active ester (injection volume between 20 and 100 µL depending on the performed glycosylation reactions) was collected, dried, and re-solubilized in phosphate buffer in the presence of carrier protein. 

The tentative glycosylation of the model protein RNase A (1 mg/mL protein concentration, in 100 mM phosphate buffer, pH 8; molar ratio ester/protein, 30:1; 37 °C; 96 h) with **20** purified by HILIC resulted in the chromatographic profile showed in Figure 1c. An improved glycosylation yield was observed and, notably, no presence of contaminant glutaric acid conjugates was detected. This result corroborated the effectiveness of the developed purification procedure and led us to focus on the optimization of glycosylation yields.

### 3.5. Glycoconjugate Synthesis 

#### 3.5.1. Optimization of Glycosylation Conditions on the Model Protein RNase A

**Figure 2:**
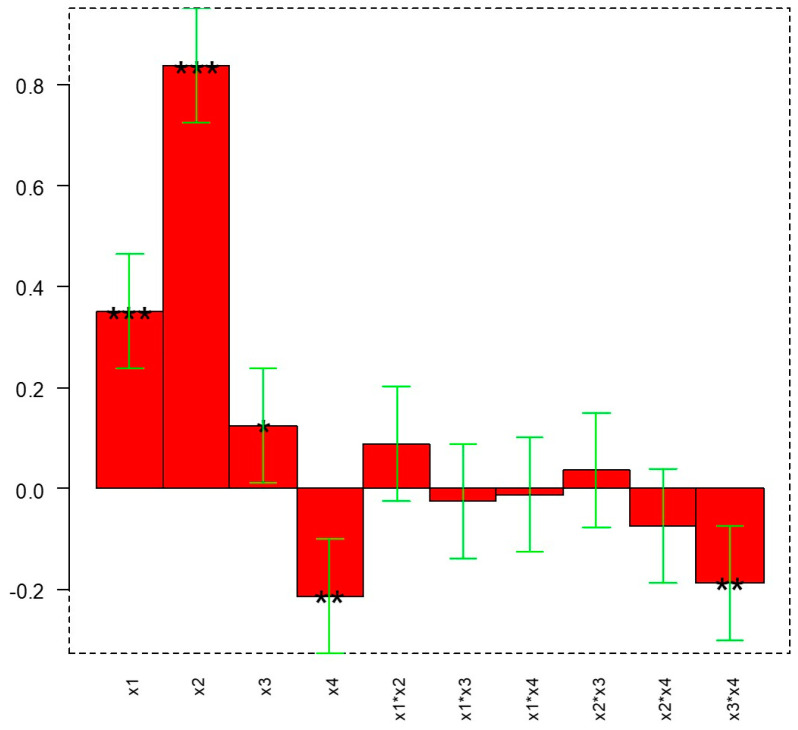
Coefficients and significance obtained in the full factorial design. The numbers on the x-axis refer to the coefficients of the terms of the models X1–X4, as defined in the text and in Table S2. The height of the boxes (*y*-axis) represents the value of each coefficient. The whiskers represent the confidence interval computed for each coefficient, and the stars refer to the *p*-value for each coefficient: * *p* ≤ 0.05, ** *p* ≤ 0.01, *** *p* ≤ 0.001. Model equation: y = 3.0 + 0.35·X1 + 0.84·X2 + 0.13·X3 − 0.21·X4 − 0.18·X3·X4.

To improve the glycosylation efficiency, we studied the effect of four experimental factors, namely the protein concentration (X1, 1 to 4 mg/mL), the active ester/protein molar ratio (X2, 50 to 100), the buffer pH (X3, 7.5 to 8.0), and the temperature (X4, 20 to 37 °C) on bound sugar/protein ratio (mol/mol). We considered the simplest glycosylation consisting of the conjugation of mannose ester **19** with the model protein RNase A, and the average number of incorporated saccharides as response. The full factorial experimental plan required 17 experiments (2^4^ experiments plus one center point). Reaction mixtures were analyzed without any further purification using HILIC chromatography, and the relative abundance of each glycoform was deduced from the corresponding peak area. Factors, limits, and results are summarized in Table S2. The coefficients (Figure 2) were statistically significant (with *p* ≤ 0.001 for X1 and X2; *p* ≤ 0.01 for X4 and *p* ≤ 0.05 for X3), and the center value of the experimental domain (Exp#17, Table S2) agreed with the measured yield (experimental: 3.1; predicted: 3.2). 

As expected, the greatest influence was exerted by X2 (mannose ester/RNase A molar ratio). A molar ratio of 100:1 (corresponding to a 10:1 mannose ester/lysine ratio) significantly increased glycosylation yields in comparison to a molar ratio of 50:1. High protein concentration also positively affects the glycosylation efficiency, probably by favoring the lysine–ester interaction. A buffer pH value of 8.0 and a lower temperature only slightly improve the glycosylation yields. Interestingly, the model showed a significant negative interaction between these two factors (*p* ≤ 0.01), suggesting that it is convenient to maintain a lower temperature while increasing the reaction pH.

Final glycosylation protocol entails a protein concentration of 4 mg/mL, a molar ratio of 100:1, buffer at pH of 8, and a temperature of 20 °C. Reaction time was fixed at 16 h since monitoring at longer reaction times did not show any further increase in the glycosylation yields.

#### 3.5.2. Study of mono-, di- and tri-Mannose Active Ester **19**, **20**, **21** Reactivity in the Glycosylation of the Model Protein RNase A

The development of an alternative purification approach and the optimization of glycosylation conditions led to define a glycosylation protocol for the preparation of highly glycosylated and pure glycoconjugates. The protocol was applied to the conjugation of **19**, **20,** and **21** to the model protein RNase A, to determine both whether and to what extent does the elongation of sugar chain affect the reactivity and influence of the glycosylation site distribution. 

HILIC analysis of the reaction mixtures allowed the relative abundance of each glycoform to be estimated, as reported in Table 1. A representative HILIC-UV profile of RNase A conjugated with **20** in optimized conditions is shown in Figure 1d, while chromatograms for mono- and tri-mannose-RNase A conjugates are available in the Supplementary Materials (Figure S3). 

Unconjugated RNase A was not detected in any of the considered reaction, suggested a 100% conversion of protein into glycosylated derivatives. Comparable glycosylation outcomes in terms of average number of incorporated saccharides were observed with di- and trisaccharide esters. Unexpectedly, a lower glycosylation yield was achieved by using **19**. This surprising result can be tentatively ascribed to a particular conformation of the anomeric linker which, in the case of monosaccharide derivatives, is less prone to condensation with the protein. Indeed, the different steric hindrance of the saccharide core could affect the spatial disposition of the linker, leading to different behaviors towards the glycosylation reaction. This experimental evidence, which goes beyond the scope of the present work, is currently under investigation.

**Figure 3:**
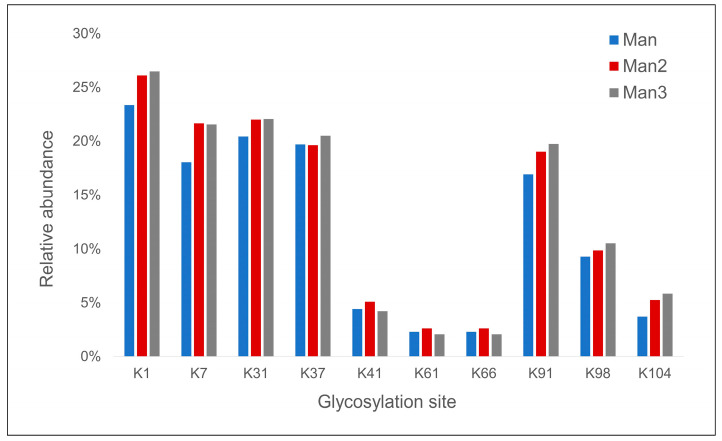
Percentage of relative abundance of each glycosylation site in the conjugation of RNase A with **19**, **20**, or **21**. Site 1 was considered to be the glycosylation site of both the N-terminal amino group and the ε-amino group of the lateral chain of K1.

The chymotryptic digestion of reaction mixtures and the HILIC-ESI-MS^3^ analysis of the resulting glycopeptides allowed to determine the relative reactivity of lysine residues of RNase A. The involvement of each lysine residue in the conjugation with **19**, **20,** and **21** was estimated by the sum of the relative glycopeptide area in MS. Percentage of relative abundances are reported in Figure 3 and show the same profile for the three esters, with the main involvement of the N-terminal portion of RNase A.

**Figure 4:**
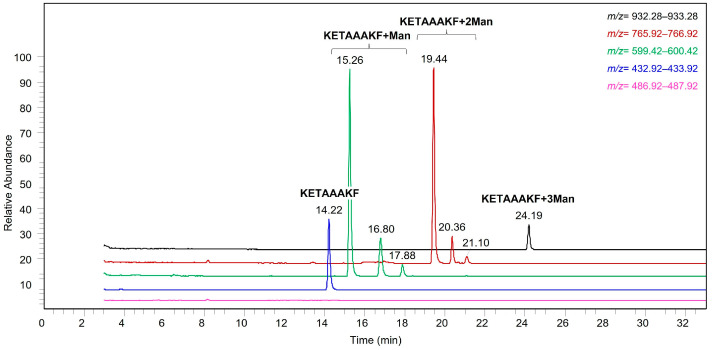
EIXs for doubly charged ions corresponding to N-terminal peptide/glycopeptide of RNase A. Pink trace: peptide conjugated to glutaric acid; blue trace: unmodified peptide; green trace: peptide conjugated to one mannose units; red trace: peptide conjugated to two mannose units; black trace: peptide conjugated to three mannose units.

The glycopeptide analysis made it possible to achieve a detailed characterization of produced glycoconjugates, and to confirm, at the peptide level, the absence of contaminant presence of glutaric acid conjugates. As an example, Figure 4 shows the extracted ion chromatograms (IEXs) of peptide/glycopeptides containing the N-terminal position of RNase A after glycosylation with **19**. The N-terminal peptide formed by chymotryptic digestion of RNase A, namely KETAAAKF, has three potential glycosylation sites: two lysine residues and the N-terminal amino group. This peptide was detected as unmodified (blue trace), mono-glycosylated (green trace), di-glycosylated (red trace), and tri-glycosylated (black trace). Both mono- and di-glycosylated species are split in three well resolved peaks that are related to the different positional isomers.

By extracting the ion current for the doubly charged ion of the hypothetical peptide formed by conjugation with glutaric acid (pink trace), no peaks are generated; this confirms that the developed glycosylation protocol successfully prevents the formation of contaminant conjugates.

#### 3.5.3. Glycosylation of Antitubercular Antigenic Proteins Ag85B wt and -dm

The achievement of high conjugation efficiency and acceptable purity is a crucial aspect in the synthesis of glycoconjugates for therapeutic use, such as glycovaccines. 

The glycosylation protocol was tested in the glycosylation of recombinant antigenic protein Ag85B wt and its variant Ag85B-dm (K30R/282R). While Ag85B has 8 lysine residues as potential glycosylation sites, the substitution of the two most reactive ones in Ag85B-dm results in six potential glycosylation sites and a reduced reactivity. Both proteins were conjugated with **20** and **21** by applying the reaction conditions previously optimized. Since mannose ester showed a lower reactivity in the study performed on RNase A, **19** was not considered in the glycosylation of antigenic carriers. For the HILIC-UV-MS analysis, the elution gradient was modified to achieve an optimal retention of Ag85B protein and resolution of its glycoconjugates. Glycosylation yields and glycoform relative abundances for the different samples are reported in Table 2.

An almost complete modification (>99.5%) of both Ag85B variants was achieved with both saccharides, and no trace of impurities derived from side reactions with residual linker was detected.

**Figure 5:**
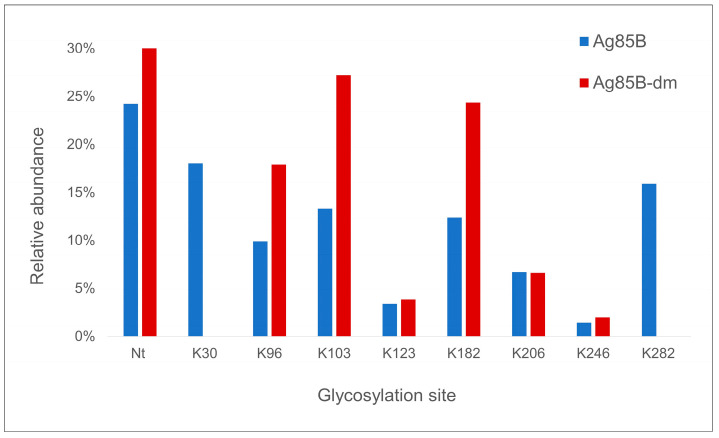
Percentage relative abundance of each glycosylation site in the conjugation of Ag85B wt and -dm with **21**.

Glycopeptide mapping analysis of conjugates revealed the predominant modification of the N-terminal site in both the wt and the dm. Percentage relative abundance of each glycosylation site in Man_3_-conjugates is reported in Figure 5. In native Ag85B, K30 and K282 were found to be the most reactive lysine residues. Their removal in Ag85B-dm increased the involvement of the less reactive sites K96, K103, and K182. On the contrary, other lysine residues (K123, K206, and K246) showed the same low reactivity in both carrier proteins.

## 4. Discussion

Conjugation via disuccinimidyl homobifunctional linkers is a common method for the synthesis of glycoconjugates. However, the well-known tendency of linkers for hydrolysis reactions represents a chemical limitation for this conjugation which may produce acid conjugates as side products. The application of the most common purification approach, consisting of the removal of excess DSG linker by washing with an organic solvent, such as EtOAc, led us to confirm the presence of a residual amount of linker in the glycosylation mixtures, which led to the formation of glutaric acid conjugates. Thus, we explored an alternative purification method based on HILIC chromatography. This approach allows the quantitative removal of the linker and the production of pure glycoconjugates. It must be underlined that only a detailed characterization, both at intact and peptide level, was allowed to verify the presence/absence of linker conjugates. Failing that, any consideration on glycoconjugate purity and identity is unreliable.

Following the optimization of the purification method, a DoE approach was used to find the reaction conditions that allow the glycan loading to be maximized. In these conditions, we studied the reactivity of mannose small-sized saccharides (**19**, **20** and **21**) in the glycosylation of RNase A. As expected, the active ester/protein molar ratio has a great influence on the glycosylation yields. In the literature, molar ratio between 30 and 150 (moles of active ester *per* mole of protein) are usually considered. In our case, a molar ratio of 100:1 was employed. The same molar ratio was used in a previous work where the RNase A was conjugated with small-sized saccharides and activated as IME thioglycosides [14]. The conjugation approach considered in this study resulted in significantly higher glycan loadings in comparison to the use of IME-activated saccharides; e.g., with Man (1–6) Man, we obtained an average incorporation number of 7.9 ± 0.1; with the same molar ratio, the average incorporation is reported as 2.3 ± 0.0 [15] for the IME disaccharide. This evidence can be explained by the high reactivity of the DSG linker and the presence of a longer spacer between the saccharide anomeric position and the reactive group, which then reduces the steric hindrance in the interaction with surface lysine residues of proteins. 

Glycopeptide mapping analysis of the resulting glycoconjugates revealed the same distribution among the potential glycosylation sites for the considered saccharides, with the N-terminal amino acid of RNase A as the predominant conjugation site. In RNase A, the N-terminal amino acid is a lysine residue, making it impossible, in principle, to discriminate between the reactivity of the main amino group and that of the amino group onto the side chain. However, it can be assumed that the reactivity of the main amino group is higher at pH 8.0. Indeed, its pKa value is lower than that of the lysine ε-amino group, thus resulting in a larger fraction of non-protonated form being available for the conjugation.

In the case of antigenic proteins, the use of a molar ratio of 100:1, ester/protein, was able to achieve satisfactory yields with <0.5% of unconjugated protein for both Ag85B wt and dm. Interestingly, no significant differences in terms of sugar loading were observed between the native and the variant protein, even if the latter is missing in the two most reactive reaction sites. In a previous work [18], the glycosylation of the same proteins via IME-saccharides showed a reduced reactivity for Ag85B-dm with a glycosylation yield of 91.5% and an average number of incorporate saccharides of 2.0 by using the disaccharide Man(1-6)Man with a molar ratio of 200:1. The glycosylation via DSG linker of the same protein with the same disaccharide and at half the molar ratio resulted in a 100% yield and in a more satisfying average number of bond sugars, i.e., 3.4.

In the case of Ag85B, we also did not observe a reduction of reactivity by increasing sugar length from di- to trisaccharide. This result is extremely relevant for the future synthesis of glycoconjugate vaccines, which require longer carbohydrate antigens. Considering that the use of more complex antigens will presumably require the sugar/protein molar ratio to be reconsidered, an accurate balance between the use of a sustainable saccharide molar excess and the achievement of an adequate glycosylation degree should be sought; this should also consider the specific antigen potency.

By glycopeptide mapping analysis, the N-terminal alanine residue was found to be the most reactive site, thus confirming the higher reactivity of the N-terminal amino group in these experimental conditions. This is a positive result in the case of recombinant Ag85B proteins, as the N-terminal region is not involved in their antigenicity [17]. After the N-terminal amino group, the most reactive sites in Ag85B wt were K30 and K282, where two lysine residues which are missing in Ag85B-dm. The same glycan loading was achieved for both Ag85B variants mainly because of the increased reactivity of three lysine residues, namely K96, K103, and K182, in Ag85B-dm. This evidence suggests a competitive mechanism among the different reactive sites and explains the great influence of molar ratio on carbohydrate loading.

Based on the achieved results, conjugation via disuccinimidyl linkers appears to be a convenient strategy in the case of the optimized Ag85B-dm carrier; future research will consider this for its glycosylation with larger-sized antigenic oligosaccharides, and in the context of the rational development of a glycoconjugate vaccine. 

## 5. Conclusions

In the present work, a common conjugation chemistry based on the use of a disuccinimidyl functional linker (namely DSG) was exploited for the synthesis of glycoconjugates. A detailed analytical characterization confirmed that the intrinsic tendency of the linker for a hydrolysis reaction limited the conjugation approach. We therefore addressed this issue by developing a novel, mostly selective, purification method based on HILIC chromatography. Once the purification protocol was set, we focused our attention on the optimization of glycosylation yield by using the model protein RNase A and the 3-aminopropyl mannose **19**. By applying the selected conditions, excellent yields were obtained in the conjugation of RNase A with newly synthesized mono-, di-, and tri-saccharide derivatives. The same protocol was then applied to the antigenic proteins Ag85B wt and -dm. The glycosylation outcome was highly satisfactory (glycosylation yields > 99.5%), even in the case of mutated carrier. 

In summary, the proposed purification method, together with a detailed structural characterization and a rational optimization of glycosylation conditions, can make this conjugation approach suitable for the synthesis of pure and high-loaded glycoconjugates, and should be considered for carbohydrate-based vaccine synthesis.

## Figures and Tables

**Table 1:** Synthesis of RNase A glycoconjugates. Percentage relative abundance of each glycoform was estimated from the UV area of the corresponding peak on HILIC chromatogram. Glycoform assignment was assessed by MS analysis.

RNase A Glycoconjugates	Number of Incorporated Saccharides (% Relative Abundance)										Saccharide Bound/RNase A
	1	2	3	4	5	6	7	8	9	10	(mol/mol) ± SD
Man	0.8 ± 0.2	4.3 ± 0.4	12.5 ± 1.3	22.2 ± 0.1	25.5 ± 0.9	19.9 ± 0.6	11.0 ± 0.5	3.5 ± 0.3	0.4 ± 0.1	-	4.9 ± 0.1
Man_2_	-	-	-	-	2.1 ± 0.5	9.2 ± 0.1	24.4 ± 0.6	34.1 ± 3.2	23.9 ± 0.5	6.3 ± 2.6	7.9 ± 0.1
Man_3_	-	-	-	-	2.6 ± 0.7	15.0 ± 2.6	35.0 ± 1.8	35.1 ± 3.1	12.3 ± 1.9	2.1 ± 0.5	7.6 ± 0.2

**Table 2:** Synthesis of Ag85B wt and -dm glycoconjugates. Percentage relative abundance of each glycoform was estimated from the UV area of the corresponding peak on HILIC chromatogram. Glycoform assignment was assessed by MS analysis.

Glycoconjugate	Number of Incorporated Saccharides (% Relative Abundance) *										Saccharide Bound/Protein
	1	0	1	2	3	4	5	6	7	8	(mol/mol) *
Ag85B-Man_2_		-	4.3	14.5	29.3	30.9	16.2	4.3	0.5	0.2	3.5
Ag85B-dm-Man_2_		-	4.8	19.6	31.3	26.3	13.4	4.0	0.6	-	3.4
Ag85B-Man_3_		0.1	8.1	19.9	25.4	26.1	14.3	4.9	1.0	0.2	3.3
Ag85B-dm-Man_3_		0.4	5.7	20.8	34.9	26.8	9.7	1.7	0.3	-	3.2

* Mean values of two replicates.

## Data Availability

The data presented in this study are available on request from the corresponding author.

## Supplementary Materials

The following supporting information can be downloaded at: https://www.mdpi.com/article/10.3390/pharmaceutics15051321/s1, Figure S1. Amino acid sequence of (a) RNase A, (b) Ag85B and (c) Ag85B-dm; Table S1. Changes in DSG and active ester (**19**, **20**) chromatographic UV areas in samples purified through precipitation with EtOAc; Figure S2. HILIC-UV profile of **21**; Table S2. Full factorial experimental plan 2^4^ with one center point and results; Figure S3. HILIC-UV profile of RNase A conjugated with (a) **19** and (b) **21** in optimized conditions; Table S3. List of glycopeptides detected in HILIC-UV-MS^3^ analysis of chymotryptic digestion of RNase A conjugated with **19**, **20** and **21**; Table S4. List of glycopeptides detected in HILIC-UV-MS^3^ analysis of chymotryptic digestion of Ag85B conjugated with **21**; Table S5. List of glycopeptides detected in HILIC-UV-MS^3^ analysis of chymotryptic digestion of Ag85B-dm conjugated with **21**; Detailed synthesis and copies of ^1^H NMR (400 MHz), ^13^C NMR and DEPT 135 spectra for compound **9**, **11**, **14**, **16** and **18**.

## References

[1] Del Bino L., Østerlid K.E., Wu D.Y., Nonne F., Romano M.R., Codée J., Adamo R. (2022). Synthetic Glycans to Improve Current Glycoconjugate Vaccines and Fight Antimicrobial Resistance. Chem. Rev..

[2] Micoli F., Adamo R., Costantino P. (2018). Protein Carriers for Glycoconjugate Vaccines: History, Selection Criteria, Characterization and New Trends. Molecules.

[3] Hoyos P., Perona A., Bavaro T., Berini F., Marinelli F., Terreni M., Hernáiz M.J. (2022). Biocatalyzed Synthesis of Glycostructures with Anti-infective Activity. Acc. Chem. Res..

[4] Smith B.R., Guo Z. (2021). Oligosaccharide Antigen Conjugation to Carrier Proteins to Formulate Glycoconjugate Vaccines. Methods Mol. Biol..

[5] Liao J., Pan B., Liao G., Zhao Q., Gao Y., Chai X., Zhuo X., Wu Q., Jiao B., Pan W. (2019). Synthesis and immunological studies of β-1,2-mannan-peptide conjugates as antifungal vaccines. Eur. J. Med. Chem..

[6] Wang J., Zhang Y., Zhu Y., Liu J., Chen Y., Cao X., Yang Y. (2020). Total Synthesis and Immunological Evaluation of the Tri-d-glycero-d-manno-heptose Antigen of the Lipopolysaccharide as a Vaccine Candidate against Helicobacter pylori. Org. Lett..

[7] Wang L., Feng S., An L., Gu G., Guo Z. (2015). Synthetic and Immunological Studies of Mycobacterial Lipoarabinomannan Oligosaccharides and Their Protein Conjugates. J. Org. Chem..

[8] Kabanova A., Adamo R., Proietti D., Berti F., Tontini M., Rappuoli R., Costantino P. (2010). Preparation, characterization and immunogenicity of HIV-1 related high-mannose oligosaccharides-CRM197 glycoconjugates. Glycoconj. J..

[9] Naini A., Bartetzko M.P., Sanapala S.R., Broecker F., Wirtz V., Lisboa M.P., Parameswarappa S.G., Knopp D., Przygodda J., Hakelberg M. (2022). Semisynthetic Glycoconjugate Vaccine Candidates against *Escherichia coli* O25B Induce Functional IgG Antibodies in Mice. JACS Au.

[10] Wang Z., Enotarpi J., Buffi G., Pezzicoli A., Gstöttner C.J., Nicolardi S., Balducci E., Fabbrini M., Romano M.R., van der Marel G.A. (2022). Chemical Synthesis and Immunological Evaluation of Fragments of the Multiantennary Group-Specific Polysaccharide of Group B. JACS Au.

[11] Zhao Y., Wang S., Wang G., Li H., Guo Z., Gu G. (2019). Synthesis and immunological studies of group A *Streptococcus* cell-wall oligosaccharide–streptococcal C5a peptidase conjugates as bivalent vaccines. Org. Chem. Front..

[12] Möginger U., Resemann A., Martin C.E., Parameswarappa S., Govindan S., Wamhoff E.C., Broecker F., Suckau D., Pereira C.L., Anish C. (2016). Cross Reactive Material 197 glycoconjugate vaccines contain privileged conjugation sites. Sci. Rep..

[13] Izumi M., Okumura S., Yuasa H., Hashimoto H. (2003). Mannose-BSA Conjugates: Comparison Between Commercially Available Linkers in Reactivity and Bioactivity. J. Carbohydr. Chem..

[14] Tanzi L., Robescu M., Marzatico S., Recca T., Zhang Y., Terreni M., Bavaro T. (2020). Developing a Library of Mannose-Based Mono- and Disaccharides: A General Chemoenzymatic Approach to Monohydroxylated Building Blocks. Molecules.

[15] Bavaro T., Filice M., Temporini C., Tengattini S., Serra I., Morelli C.F., Massolini G., Terreni M. (2014). Chemoenzymatic synthesis of neoglycoproteins driven by the assessment of protein surface reactivity. RSC Adv..

[16] Bavaro T., Tengattini S., Piubelli L., Mangione F., Bernardini R., Monzillo V., Calarota S., Marone P., Amicosante M., Pollegioni L. (2017). Glycosylation of Recombinant Antigenic Proteins from *Mycobacterium tuberculosis*: In Silico Prediction of Protein Epitopes and Ex Vivo Biological Evaluation of New Semi-Synthetic Glycoconjugates. Molecules.

[17] Piubelli L., Campa M., Temporini C., Binda E., Mangione F., Amicosante M., Terreni M., Marinelli F., Pollegioni L. (2013). Optimizing Escherichia coli as a protein expression platform to produce *Mycobacterium tuberculosis* immunogenic proteins. Microb. Cell Fact..

[18] Rinaldi F., Tengattini S., Piubelli L., Bernardini R., Mangione F., Bavaro T., Paone G., Mattei M., Pollegioni L., Filice G. (2018). Rational design, preparation and characterization of recombinant Ag85B variants and their glycoconjugates with T-cell antigenic activity against. RSC Adv..

[19] Rinaldi F., Lupu L., Rusche H., Kukačka Z., Tengattini S., Bernardini R., Piubelli L., Bavaro T., Maeser S., Pollegioni L. (2019). Epitope and affinity determination of recombinant *Mycobacterium tuberculosis* Ag85B antigen towards anti-Ag85 antibodies using proteolytic affinity-mass spectrometry and biosensor analysis. Anal. Bioanal. Chem..

[20] Armarego W.L.F., Chai C.L.L. (2013). Purification of Laboratory Chemicals.

[21] Cramer J., Aliu B., Jiang X., Sharpe T., Pang L., Hadorn A., Rabbani S., Ernst B. (2021). Poly-l-lysine Glycoconjugates Inhibit DC-SIGN-mediated Attachment of Pandemic Viruses. ChemMedChem.

[22] Zhang P., Ma J., Zhang Q., Jian S., Sun X., Liu B., Nie L., Liu M., Liang S., Zeng Y. (2019). Monosaccharide Analogues of Anticancer Peptide R-Lycosin-I: Role of Monosaccharide Conjugation in Complexation and the Potential of Lung Cancer Targeting and Therapy. J. Med. Chem..

[23] Tanzi L., Rubes D., Bavaro T., Sollogoub M., Serra M., Zhang Y., Terreni M. (2022). Controlled Decoration of [60]Fullerene with Polymannan Analogues and Amino Acid Derivatives through Malondiamide-Based Linkers. Molecules.

[24] Tengattini S., Domínguez-Vega E., Temporini C., Bavaro T., Rinaldi F., Piubelli L., Pollegioni L., Massolini G., Somsen G.W. (2017). Hydrophilic interaction liquid chromatography-mass spectrometry as a new tool for the characterization of intact semi-synthetic glycoproteins. Anal. Chim. Acta.

