# Supplementary material for: Glycovaccine Design: Optimization of Model and Antitubercular Carrier Glycosylation via Disuccinimidyl Homobifunctional Linker
Source: Pharmaceutics. 2023 Apr 23;15(5):1321. doi: 10.3390/pharmaceutics15051321 (PMC10223089; doi:10.3390/pharmaceutics15051321)

Glycovaccine design: optimization of model and antitubercular carrier glycosylation *via* disuccinimidyl homobifunctional linker

Table of Contents

S2 **Figure S1.** Amino acid sequence of (a) RNase A, (b) Ag85B, and (c) Ag85B-dm.

S3 **Table S1.** Changes in DSG and active ester (**19**, **20**) chromatographic peak UV areas in samples purified through precipitation with ethyl acetate.

Figure S2. HILIC-UV profile of **21**.

S4 **Table S2.** Full factorial experimental plan 2^4 with one center point and results.

Figure S3. HILIC-UV profile of RNase A conjugated with (a) **19** and (b) **21** in optimized conditions.

S5 **Table S3.** List of glycopeptides detected in HILIC-UV-MS^3 analysis of chymotryptic digestion of RNase A conjugated with **19**, **20** and **21.**

S6 **Table S4.** List of glycopeptides detected in HILIC-UV-MS^3 analysis of chymotryptic digestion of Ag85B conjugated with **21**.

S7 **Table S5.** List of glycopeptides detected in HILIC-UV-MS^3 analysis of chymotryptic digestion of Ag85B-dm conjugated with **21**.

S8 Detailed synthesis and copies of ^1H NMR (400 MHz), ^{13}C NMR and DEPT 135 spectra for compound **9.**

S10 Detailed synthesis and copies of ^1H NMR (400 MHz), ^{13}C NMR and DEPT 135 spectra for compound **11.**

S12 Detailed synthesis and copies of ^1H NMR (400 MHz), ^{13}C NMR and DEPT 135 spectra for compound **14.**

S14 Detailed synthesis and copies of ^1H NMR (400 MHz), ^{13}C NMR and DEPT 135 spectra for compound **16.**

S16 Detailed synthesis and copies of ^1H NMR (400 MHz), ^{13}C NMR and DEPT 135 spectra for compound **18.**

Figure S1 Amino acid sequence of (a) RNase A, (b) Ag85B and (c) Ag85B-dm.

(a)

```
        10         20         30         40         50
KETAAAKFER QHMDSSTSAA SSSNYCNQMM KSRNLTKDRC KPVNTFVHES
        60         70         80         90        100
LADVQAVCSQ KNVACKNGQT NCYQSYSTMS ITDCRETGSS KYPNCAYKTT
       110        120
QANKHIIVAC EGNPYVPVHF DASV
```

(b)

```
        10         20         30         40         50
AMAISDPFSR PGLPVEYLQV PSPSMGRDIK VQFQSGGNNS PAVYLLDGLR
        60         70         80         90        100
AQDDYNGWDI NTPAFEWYYQ SGLSIVMPVG GQSSFYSDWY SPACGKAGCQ
       110        120        130        140        150
TYKWETFLTS ELPQWLSANR AVKPTGSAAI GLSMAGSSAM ILAAYHPQQF
       160        170        180        190        200
IYAGSLSALL DPSQGMGPSL IGLAMGDAGG YKAADMWGPS SDPAWERNDP
       210        220        230        240        250
TQQIPKLVAN NTRLWVYCGN GTPNELGGAN IPAEFLENFV RSSNLKFQDA
       260        270        280        290
YNAAGGHNAV FNFPPNGTHS WEYWGAQLNA MKGDLQSSLG AG
```

(c)

```
        10         20         30         40         50
AMAISDPFSR PGLPVEYLQV PSPSMGRDIR VQFQSGGNNS PAVYLLDGLR
        60         70         80         90        100
AQDDYNGWDI NTPAFEWYYQ SGLSIVMPVG GQSSFYSDWY SPACGKAGCQ
       110        120        130        140        150
TYKWETFLTS ELPQWLSANR AVKPTGSAAI GLSMAGSSAM ILAAYHPQQF
       160        170        180        190        200
IYAGSLSALL DPSQGMGPSL IGLAMGDAGG YKAADMWGPS SDPAWERNDP
       210        220        230        240        250
TQQIPKLVAN NTRLWVYCGN GTPNELGGAN IPAEFLENFV RSSNLKFQDA
       260        270        280        290
YNAAGGHNAV FNFPPNGTHS WEYWGAQLNA MRGDLQSSLG AG
```

Table S1. Changes in DSG and active ester (**19**, **20**) chromatographic peak UV areas in samples purified through precipitation with ethyl acetate.

Compound	EtOAc washes	Molar ratio DSG: activated ester	Loss (%)
**19**	Pre-purification	15:1	-
	9-volume	2.6:1	35
	19-volume	2.0:1	49
**20**	Pre-purification	15:1	-
	9-volume	1.4:1	24
	19-volume	0.4:1	35

Figure S2. HILIC-UV profile of **21**. Detection was performed at 214 nm. For other experimental details see manuscript, Section 2.3.4.

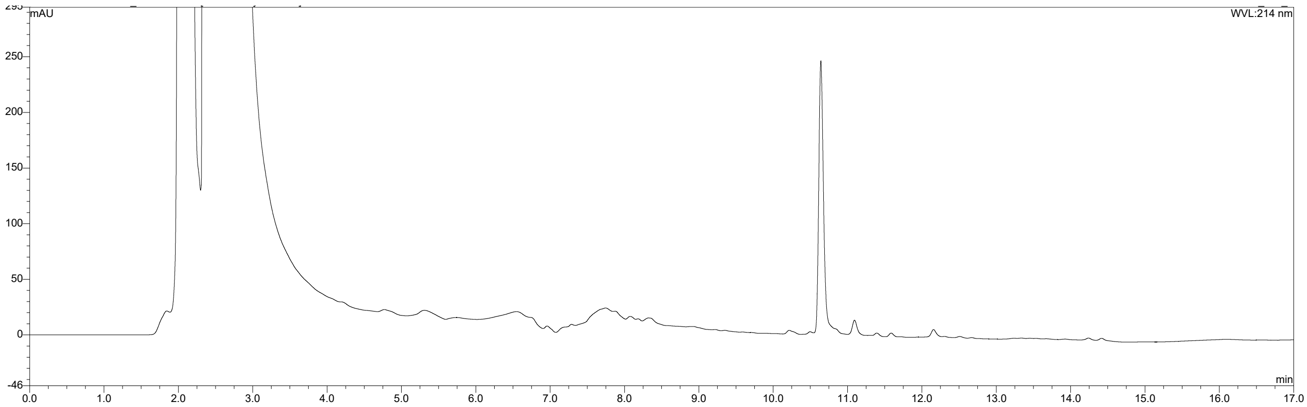

Table S2. Full factorial experimental plan 2^4 with one center point and results.

Exp#	X1 (RNase A concentration)	X2 (19/RNase A, mol/mol)	X3 (Buffer pH)	X4 (Temperature, °C)	Y (Mannose bound/RNase A, mol/mol)
1	1	50:1	7.5	20	1.8
2	4	50:1	7.5	20	2.4
3	1	100:1	7.5	20	3.3
4	4	100:1	7.5	20	4.1
5	1	50:1	8.0	20	2.2
6	4	50:1	8.0	20	2.8
7	1	100:1	8.0	20	4.1
8	4	100:1	8.0	20	5.0
9	1	50:1	7.5	37	1.7
10	4	50:1	7.5	37	2.4
11	1	100:1	7.5	37	3.2
12	4	100:1	7.5	37	4.1
13	1	50:1	8.0	37	1.9
14	4	50:1	8.0	37	2.1
15	1	100:1	8.0	37	3.0
16	4	100:1	8.0	37	3.9
17	2	75:1	7.75	20	3.1

Figure S3. HILIC-UV profile of RNase A conjugated with (a) **19** and (b) **21** in optimized conditions. Detection was performed at 214 nm. Numbers indicate the incorporated sugar units.

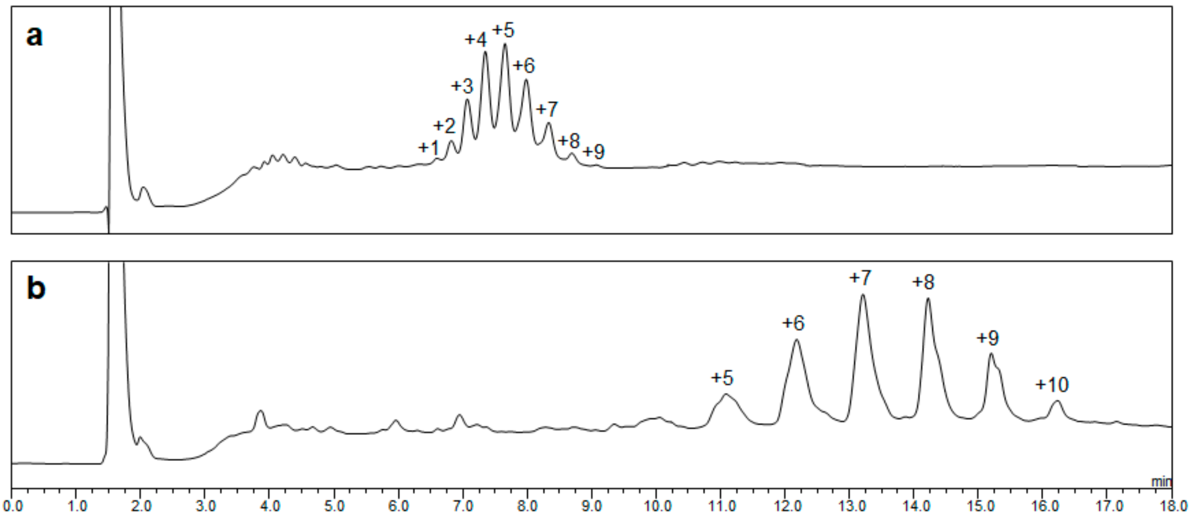

Table S3: List of glycopeptides detected in HILIC-UV-MS[3] analysis of chymotryptic digestion of RNase A conjugated with **19**, **20,** and **21.**

First aa	*Last aa*	*Number of glycosylations*	*Glycosylation site*	*Rt (min)*	*A %*	*Rt (min)*	*A %*	*Rt (min)*	*A %*
80	91	1	91	15.20	1.7	19.99	2.4	23.41	1.5
1	8	1	1	15.29	6.7	20.99	1.4	24.50	3.0
			7	16.80	3.9	22.04	0.7	25.01	1.7
			1	17.90	1.0	22.88	0.1	26.25	1.4
30	35	1	31	16.06	11.5	21.34	4.0	24.90	1.5
31	35	1	31	17.57	0.7	22.88	1.8	26.37	4.9
26	35	1	31	18.67	8.2	23.22	16.2	26.29	15.7
36	46	1	37	20.50	18.4	24.79	16.1	27.48	17.0
			41	21.24	3.2	25.52	1.5	28.11	0.8
98	124	1	98	23.35	8.0	26.99	6.8	28.83	7.4
			104	23.71	2.4	28.13	2.2	30.21	2.7
80	97	1	91	23.31	10.2	27.15	12.1	29.46	11.7
1	8	2	1 and 7	19.48	12.6	27.41	17.2	32.54	16.7
77	97	1	91	23.84	5.1	27.52	4.5	29.7	6.6
36	46	2	37 and 41	23.05	1.3	29.79	3.6	34.03	3.5
98	124	2	98 and 104	24.81	1.3	30.32	3.1	33.66	3.1
1	8	3	1 (x2) and 7	24.21	1.5	33.56	3.7	39.29	3.2
59	73	2	61 and 67	23.15	2.3	27.53	2.6	29.68	2.1

Table S4: List of glycopeptides detected in HILIC-UV-MS^3 analysis of chymotryptic digestion of Ag85B conjugated with **21.**

Detected m/z	*z*	*Number of glycosylations*	*First aa*	*Last aa*	*Glycosylation site*	*Rt (min)*	*Area*	*A %*
1254.72	2	1	1	17	N-t	21.5	1.04E+06	13.2
1188.66	2	1	275	292	282	27.25	8.85E+05	11.2
1179.3	3	1	18	44	30	29.37	8.41E+05	10.7
755.20	2	1	1	8	N-t	20.48	5.08E+05	6.4
1198.08	2	1	180	195	182	27.41	4.27E+05	5.4
1218.8	2	1	103	116	103	22.2	4.17E+05	5.3
1517.88	3	1	74	108	96	29.33	3.06E+05	3.9
1086.02	2	1	117	132	123	27.25	2.68E+05	3.4
1331.34	3	1	262	292	282	28.43	2.34E+05	3.0
684.62	2	1	103	107	103	21.73	1.92E+05	2.4
1511.74	3	1	9	44	30	30.12	1.85E+05	2.3
1385.2	3	1	87	116	103	26.15	1.78E+05	2.3
1789.88	3	1	59	101	96	28.55	1.72E+05	2.2
1184.54	2	1	173	189	182	26.61	1.52E+05	1.9
1077.18	2	1	202	214	206	24.05	1.42E+05	1.8
1845.94	3	1	174	217	206	28.22	1.40E+05	1.8
1117.84	2	1	4	17	N-t	21.77	1.38E+05	1.7
845.84	3	1	200	215	206	24.86	1.33E+05	1.7
951.96	3	1	272	292	282	26.65	1.16E+05	1.5
1976.00	4	2	167	229	182 and206	32.46	1.14E+05	1.4
820.84	3	1	18	33	30	24.9	1.09E+05	1.4
1737.07	3	2	74	108	96 and103	33.67	1.05E+05	1.3
1274.00	4	1	4	44	N-t	29.2	1.05E+05	1.3
1017.20	2	1	98	108	103	21.47	1.01E+05	1.3
1136.18	3	1	176	201	182	26.73	9.89E+04	1.3
1249.76	3	1	74	102	96	27.37	8.53E+04	1.1
1061.50	2	1	5	17	N-t	23.21	8.52E+04	1.1
1661.12	3	1	5	44	N-t and30	29.83	8.47E+04	1.1
1283.64	3	1	156	187	182	29.07	7.57E+04	1.0
1008.74	3	1	240	261	246	28.59	6.97E+04	0.9
1003.74	2	1	90	102	96	26.85	5.97E+04	0.8
1604.28	3	2	87	116	96 and103	32.05	5.47E+04	0.7
690.04	2	1	182	187	182	22.43	5.45E+04	0.7
1438.34	4	2	4	44	N-t and30	33.3	5.44E+04	0.7
1879.94	3	2	5	44	30	34.15	4.46E+04	0.6
1043.28	2	1	240	251	246	27.01	4.15E+04	0.5
1051.46	2	1	174	187	182	24.33	4.02E+04	0.5
946.76	2	1	280	292	282	25.84	2.13E+04	0.3
911.46	2	1	182	192	182	26.77	1.35E+04	0.2

Table S5: List of glycopeptides detected in HILIC-UV-MS^3 analysis of chymotryptic digestion of Ag85B-dm conjugated with **21.**

Detected m/z	*z*	*Number of glycosylations*	*First aa*	*Last aa*	*Glycosylation site*	*Rt (min)*	*Area*	*A %*
1198.22	2	1	180	195	182	27.46	7.66E+05	13.9
1253.78	2	1	1	17	N-t	21.58	6.94E+05	12.6
1517.92	3	1	74	108	96	29.41	6.40E+05	11.6
1218.28	2	1	103	116	103	22.27	6.00E+05	10.9
1017.20	2	1	98	108	103	21.8	5.16E+05	9.4
755.20	2	1	1	8	N-t	20.55	3.44E+05	6.2
684.62	2	1	103	107	103	21.8	2.40E+05	4.4
1085.92	2	1	117	132	123	27.39	2.28E+05	4.1
1184.54	2	1	173	189	182	26.71	1.92E+05	3.5
1440.08	3	1	1	33	N-t	24.67	1.87E+05	3.4
1076.36	2	1	202	214	206	24.14	1.78E+05	3.2
794.18	2	1	94	102	96	21.69	1.78E+05	3.2
1117.84	2	1	4	17	N-t	21.84	1.34E+05	2.4
1384.94	3	1	87	116	103	26.22	1.34E+05	2.4
1135.86	3	1	176	201	182	26.83	1.33E+05	2.4
1282.22	3	1	6	33	N-t	25.08	1.10E+05	2.0
1737.07	3	2	74	108	96 and103	33.71	1.02E+05	1.8
1284.00	3	1	156	187	182	29.16	9.98E+04	1.8
1844.66	3	1	174	217	206	28.28	9.73E+04	1.8
1250.08	3	1	74	102	96	27.42	8.37E+04	1.5
690.04	2	1	182	187	182	22.49	8.30E+04	1.5
1133.54	3	1	10	33	N-t	25.28	7.88E+04	1.4
1008.74	3	1	240	261	246	28.69	7.14E+04	1.3
1976.00	4	2	167	229	182 and206	32.51	6.31E+04	1.1
1050.78	2	1	174	187	182	24.41	5.92E+04	1.1
845.84	3	1	200	215	206	24.5	5.38E+04	1.0
1043.24	2	1	240	251	246	27.12	4.75E+04	0.9
1060.66	2	1	5	17	N-t	23.25	4.53E+04	0.8
837.68	2	1	9	17	N-t	23.17	3.39E+04	0.6
1003.74	2	1	90	102	96	26.75	2.50E+04	0.5
911.46	2	1	182	192	182	26.88	2.47E+04	0.4
1604.18	3	2	87	116	96 and103	32.07	2.23E+04	0.4
1789.88	3	1	59	101	96	29.24	1.21E+04	0.2

General remarks

NMR: All 1D and 2D NMR spectra were acquired using the standard pulse sequences available with Bruker Topspin 3.6 software package. Chemical shifts (δ) are given in ppm and were referenced to the solvent signals. Signal multiplicities are abbreviated as follows: s, singlet; d, doublet; t, triplet; q, quartet; dd, doublet of doublets; dt, doublet of triplets; td, triplet of doublets; m, multiplet.

HRMS: The SCIEX OS software 2.1.6 was used as an operating platform. For MS detection the following parameters were applied: Curtain gas 30 psi, Ion source gas 1 45 psi, Ion source gas 2 55 psi, Temperature 450 °C, Polarity positive, Ion spray voltage 5500 V, TOF mass range 50–2800 Da, declustering potential 60 V and collision energy 10 V.

Azidopropyl-2′,3′,4′,6′-tetra-O-acetyl-*α*-D-mannopyranosyl-(1→6)-2,3,4-tri-O-acetyl-*α*-D-mannopyranoside (9)

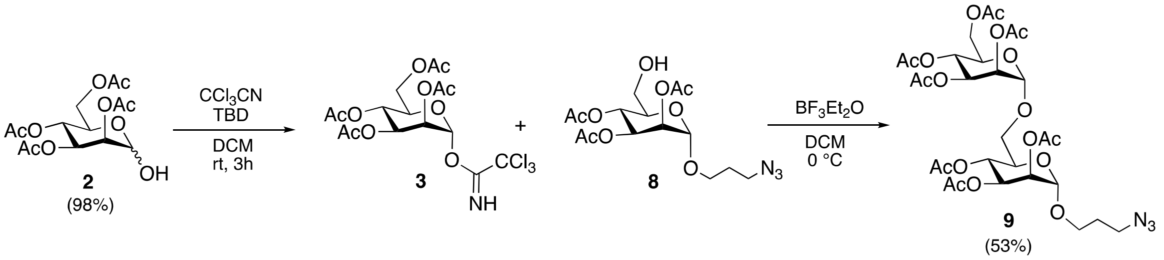

A solution of compound **2** (90 mg, 0.258 mmol, 1 eq.) in DCM (0.2 M) was added with trichloroacetonitrile (104 µL, 1.034 mmol, 4 eq.) and polystyrene supported 1,5,7-triazabicyclo[4.4.0]dec-5-ene (TBD) (0.4 eq.). The mixture was stirred at rt under nitrogen atmosphere for 4h.
Then, TBD resin was filtered off, and the organic phase was concentrated in vacuo to give **3** (colorless oil) which was used in the next synthetic step without further purifications.
The reaction was monitored by TLC (ethyl acetate /hexane 1:1).
Compound **3** was solubilized in DCM (0.1M) together with **8** (80mg, 0.205 mmol, 0.8 eq.), the solution was cooled at 0°C and BF_3Et_2O (26 µL, 0.205 mmol, 0.8 eq.) was added dropwise.
The reaction mixture was monitored by TLC (ethyl acetate/n-hexane 7:3) and purified by flash chromatography (ethyl acetate/n-hexane 6:4) to give compound **9** as a white solid (98mg, y: 53% over 2 steps).

^{1}H NMR ($CDCl_3$, 400 MHz) δ 1.89 – 1.97 (m, 2H), 1.99 (s, 3H), 2.01 (s, 3H), 2.06 (s, 3H), 2.07 (s, 3H), 2.12 (s, 3H), 2.17 (s, 6H), 3.46 (t, *J* = 6.6 Hz, 2H), 3.52 – 3.59 (m, 2H), 3.76 – 3.88 (m, 2H), 3.95 (m, 1H), 4.09 (m, 1H), 4.16 (dd, *J* = 2.2, 12.2 Hz, 1H), 4.26 (dd, *J* = 5.4, 12.2 Hz, 1H), 4.80 (d, *J* = 1.4 Hz, 1H), 4.86 (d, *J* = 1.5 Hz, 1H), 5.19 – 5.38 (m, 6H); ^{13}C{^{1}H} NMR ($CDCl_3$, 100 MHz) δ 20.6, 20.7 (4C), 20.8, 20.9, 28.6 (t), 48.2 (t), 62.4 (t), 64.9 (t), 66.0, 66.5 (2C, one carbon is a triplet), 68.6, 68.9, 69.1, 69.4 (3), 97.3, 97.4, 169.7 (s), 169.8 (s), 169.9 (2C, s), 170.0 (s), 170.1 (s), 170.6 (s); HRMS (ESI) calculated for $C_{29}H_{41}N_3O_{18}Na$ $[M+Na]^+$ 742.2277, found 742.2273 (Δ = –0.6 ppm).

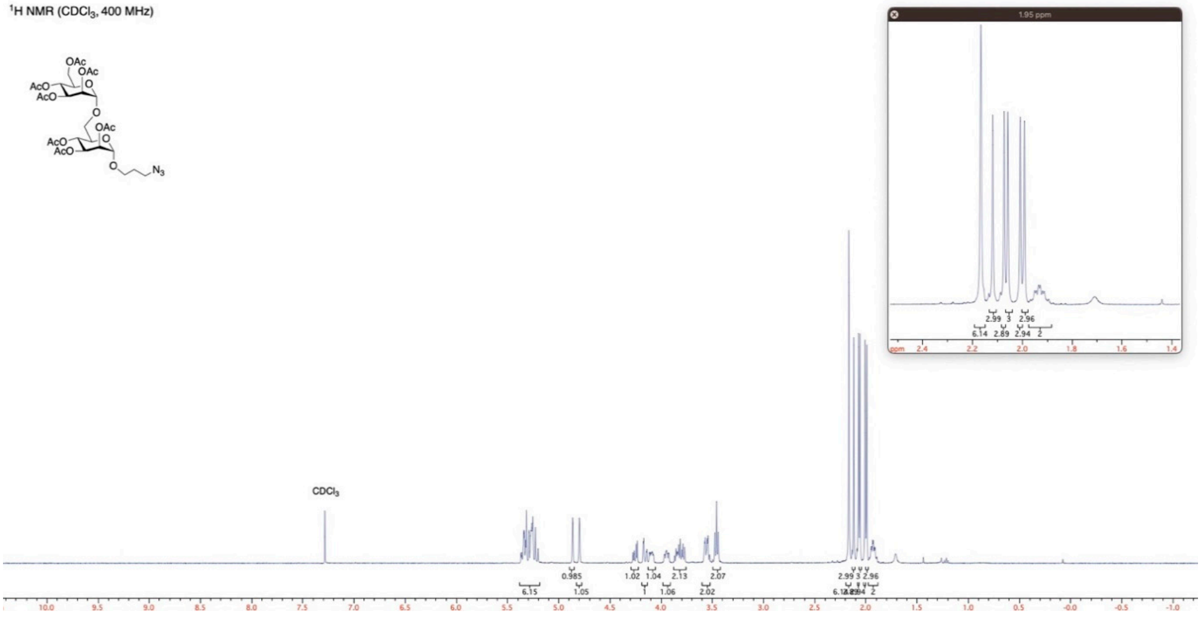

^{1}H NMR ($CDCl_3$, 400 MHz)
OAc
AcO
N$_3$
$CDCl_3$
1.95 ppm

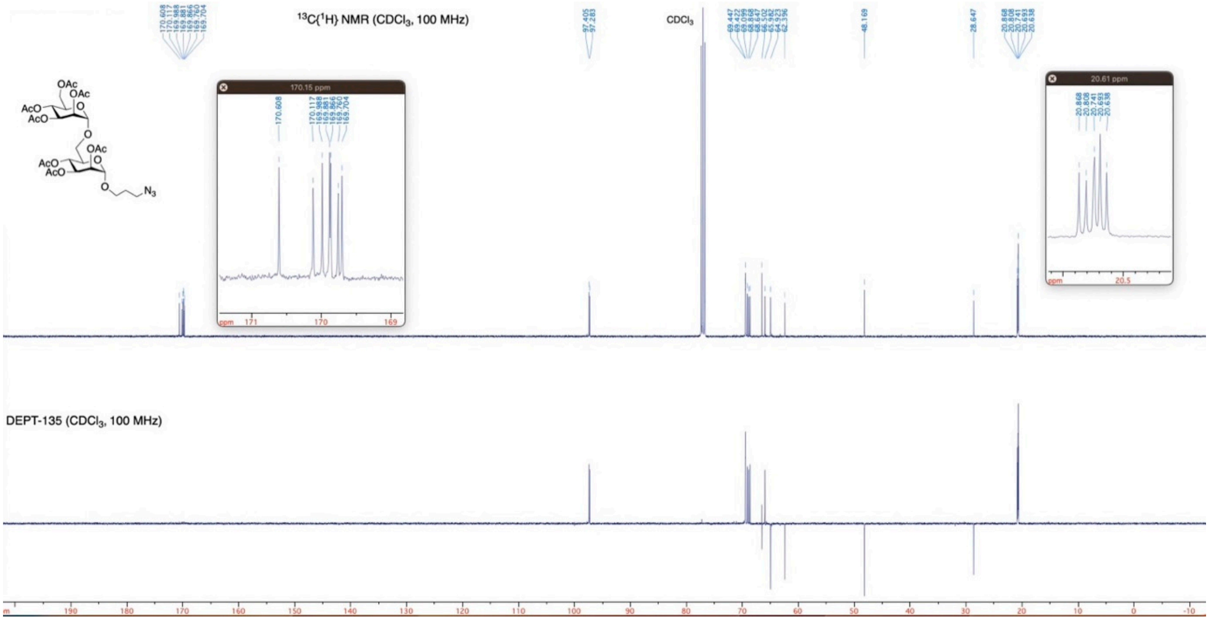

^{13}C{^{1}H} NMR ($CDCl_3$, 100 MHz)
$CDCl_3$
170.15 ppm
20.61 ppm
DEPT-135 ($CDCl_3$, 100 MHz)
OAc
AcO
N$_3$

3-Aminopropyl α-D-mannopyranosyl-(1→6)-α-D-mannopyranoside (11)

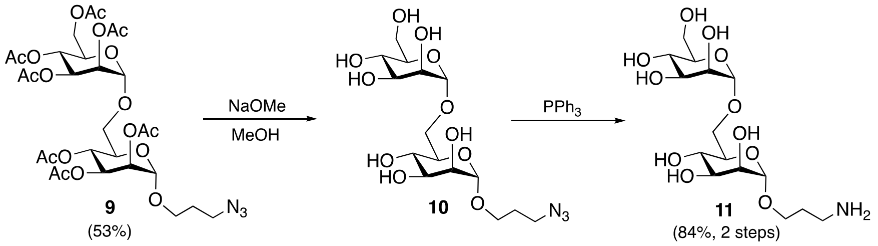

A solution of **9** (40mg, 0.056 mmol, 1 eq.) in MeOH (0.05M) was cooled at 0°C and added with NaOMe (4.5mg, 0.083 mmol, 1.5 eq.).

The reaction was stirred under nitrogen atmosphere until completion (checked by MS analysis), then acidic Dowex resin (Dowex 50w x8) was added in proportion until a pH of 6-7 was reached.

The resin was filtered off, washed with methanol, and the organic phase was evaporated, leaving intermediate **10,** which was used in the next synthetic step without further purification.

In a round bottom flask, triphenylphosphine (26 mg, 0.100 mmol, 1.8eq.) was added to a solution of **10** in THF-H_2O (85:15, 0.05M) under stirring. The reaction was heated at 75°C and monitored by MS analysis.

The THF was than evaporated, the mixture diluted with 500 µL of water, and the solid precipitate filtered off. The filtrate was evaporated to dryness affording **11** (18.7mg, y: 84%, over 2 steps).

^{1}H NMR (D_2O, 400 MHz) δ 1.69 – 1.78 (m, 2H), 2.73 – 2.87 (m, 2H), 3.42 (m, 1H), 3.47 – 3.69 (m, 9H), 3.72 (dd, *J* = 1.6, 11.8 Hz, 1H), 3.76 – 3.82 (m, 3H), 4.67 (d, *J* = 1.7 Hz, 1H), 4.72 (d, *J* = 1.7 Hz, 1H); $^{13}C\{^{1}H\}$ NMR (D_2O, 100 MHz) δ 23.1, 28.0 (t), 37.5 (t), 60.8 (t), 65.1 (t), 65.5 (t), 66.4, 66.6, 69.8, 70.5, 70.7, 70.8, 72.6, 99.4, 99.8; HRMS (ESI) calculated for $C_{15}H_{30}NO_{11}$ [M+H]$^{+}$ 400.1813, found 400.1810 (Δ = -0.8 ppm).

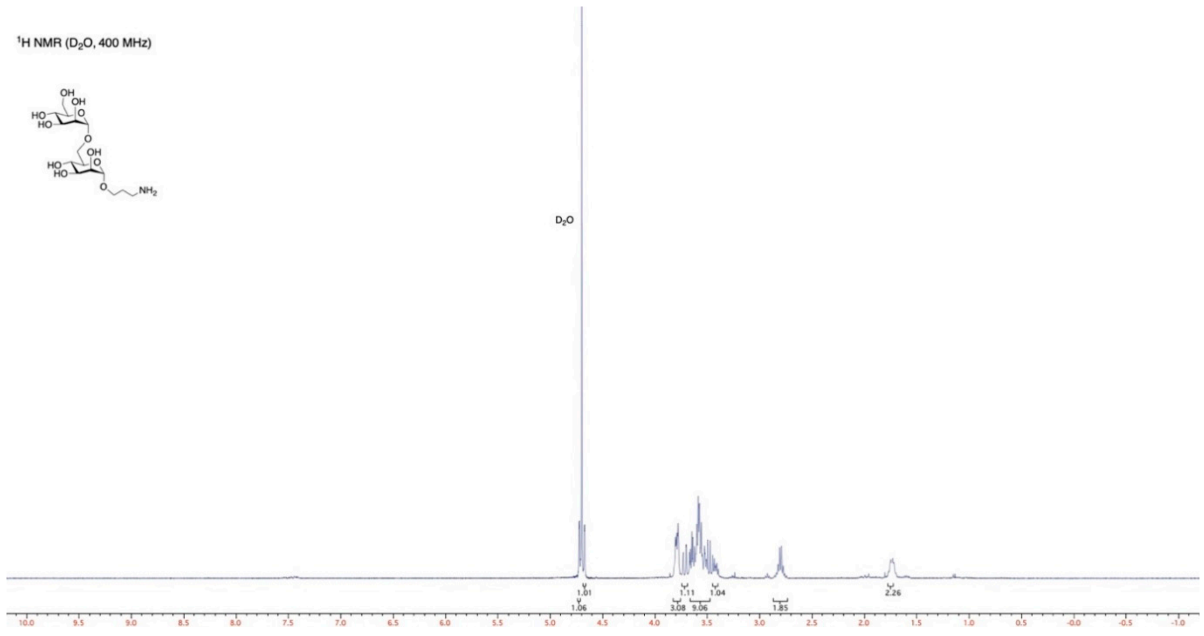
1H NMR (D2O, 400 MHz)
D2O

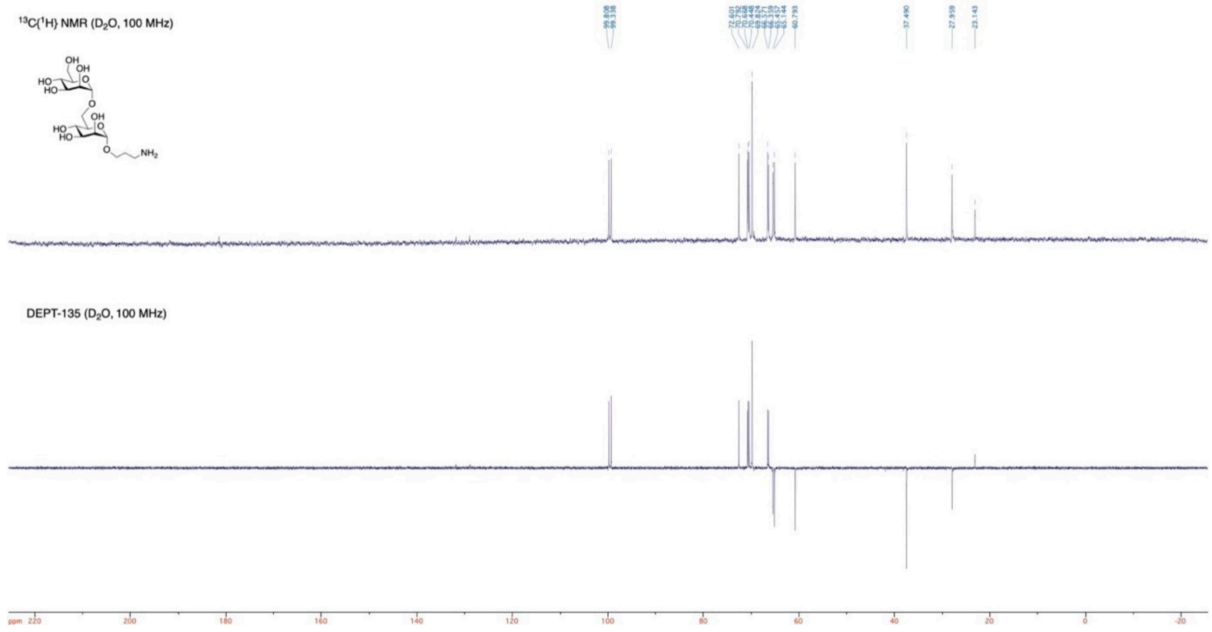
13C{1H} NMR (D2O, 100 MHz)
DEPT-135 (D2O, 100 MHz)

2′,3′,4′,6′-tetra-O-acetyl-α-D-mannopyranosyl-(1→6)-2,3,4-tri-O-acetyl- -D- mannopyranose (14)

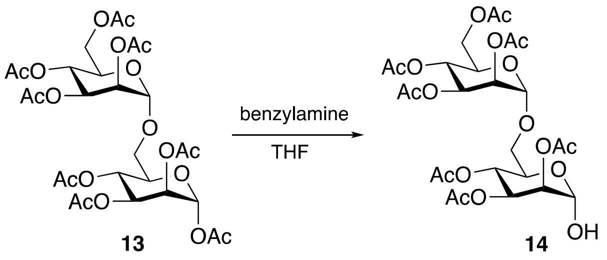

Benzylamine (29µl, 0.265 mmol, 1.5 eq.) was added to a solution of **13** (120 mg, 0.177 mmol, 1 eq.) in THF (0.1M), and the reaction mixture was stirred at rt for 48 hours. The reaction mixture was monitored by TLC (ethyl acetate/n-hexane 6:4) and purified by flash chromatography (DCM/acetone 85:15) affording **14** as a white amorphous solid (110mg, Y: 98%).

^{1}H NMR ($CDCl_3$, 400 MHz) δ 1.99 (s, 3H), 2.00 (s, 3H), 2.06 (s, 3H), 2.07 (s, 3H), 2.12 (s, 3H), 2.16 (s, 3H, 3H), 3.60 (dd, *J* = 2.6, 11.3 Hz, 1H), 3.78 (dd, *J* = 5.9, 11.3 Hz, 1H), 4.07 (m, 1H), 4.12-4.27 (m, 4H), 4.85 (d, J = 1.5 Hz, 1H), 5.20 – 5.30 (m, 5H), 5.37 (dd, *J* = 3.3, 10.1 Hz, 1H), 5.43 (dd, *J* = 3.3, 10.0 Hz, 1H); ^{13}C{^{1}H} NMR ($CDCl_3$, 100 MHz) δ 20.7 (3C), 20.8 (3C), 62.4 (t), 66.1, 66.8, 67.6 (t), 68.5, 68.8, 69.0, 69.1, 69.4, 70.0, 92.1, 97.8, 169.8 (s), 169.9 (3C, s), 170.1 (s), 170.2 (s), 170.9 (s).

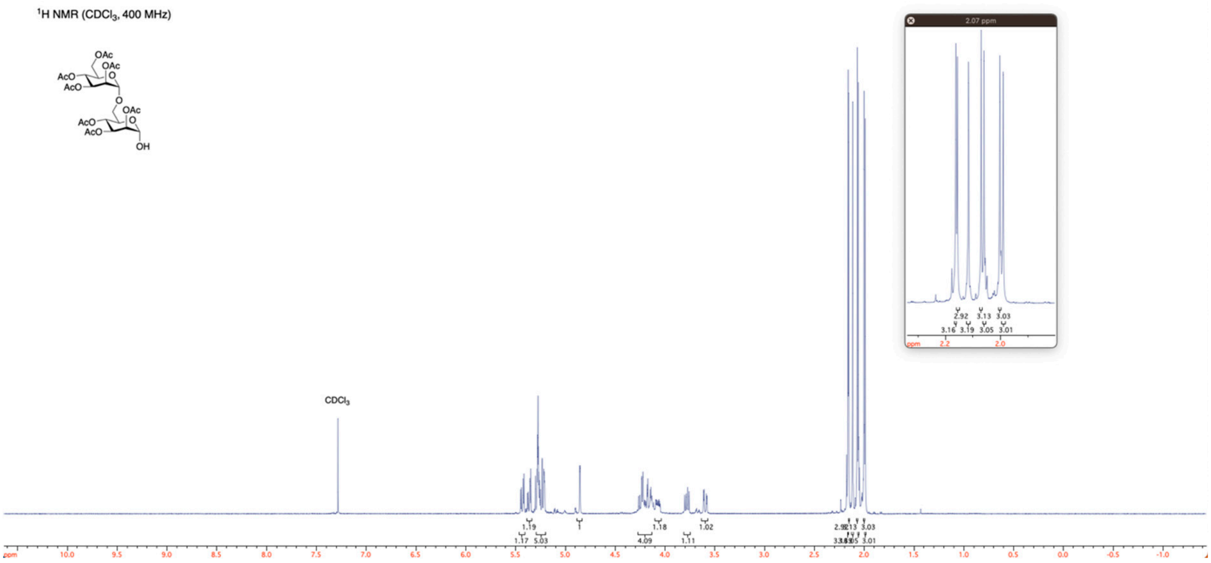

^{1}H NMR ($CDCl_3$, 400 MHz)
OAc
OAc
AcO
AcO
O
O
OAc
AcO
AcO
O
OH
$CDCl_3$
2.07 ppm

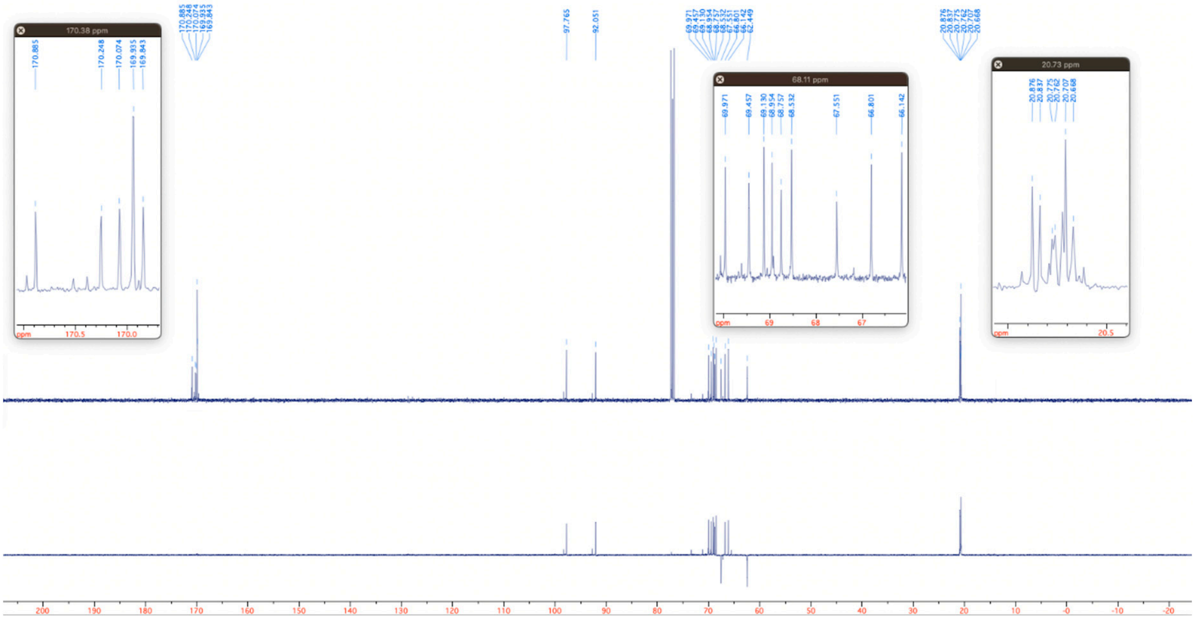

170.38 ppm
68.11 ppm
20.73 ppm
97.765
92.051

3-Azidopropryl 2″,3″,4″,6″-tetra-O-acetyl-α-D-mannopyranosyl-(1→6)-2′,3′,4′-tri-O-acetyl-α-D-mannopyranosyl-(1→6)-2,3,4-tri-O-acetyl-α-D-mannopyranoside (16)

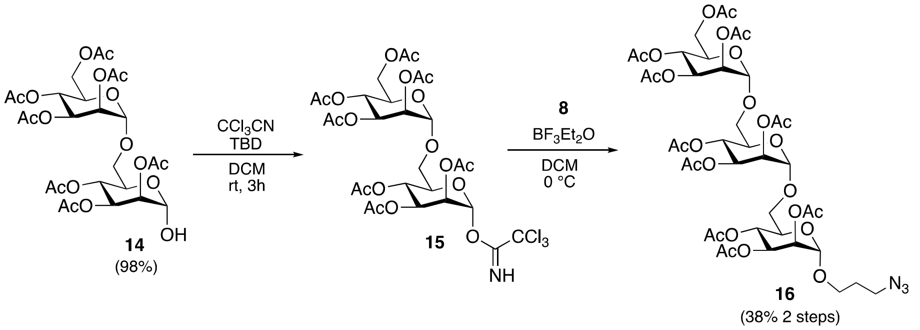

A solution of **14** (120 mg, 0.188 mmol, 1 eq.) in DCM (0.2 M) was added to trichloroacetonitrile (76 µL, 0.754 mmol, 4 eq.) and TBD (0.4 eq.). The mixture was stirred at rt, under nitrogen atmosphere, for 4h. Then, the resin was filtered off and the organic phase concentrated in vacuo to give **15** (colorless oil), which was used in the next synthetic step without further purifications.

The reaction was monitored by TLC (ethyl acetate /hexane 6:4).

The obtained oil was solubilized in DCM (0.1M) together with **8** (55mg, 0.142 mmol, 0.8 eq.), the solution was cooled at 0°C, and BF_3Et_2O (18 µL, 0.142 mmol, 0.8 eq.) was added dropwise.

The reaction mixture was monitored by TLC (ethyl acetate/n-hexane 7:3) and purified by flash chromatography (ethyl acetate/n-hexane 6:4) to obtain compound **16** as a white solid (55mg, y: 38% over 2 passages).

^{1}H NMR ($CDCl_3$, 400 MHz) δ 1.89 – 1.96 (m, 2H), 1.97 (s, 3H), 1.98 (s, 3H), 2.00 (s, 3H), 2.04 – 2.08 (m, 9H), 2.11 (s, 3H), 2.15 (s, 3H), 2.16 (s, 6H) 3.45 (t, *J* = 6.6 Hz, 2H), 3.50 – 3.60 (m, 3H), 3.74 – 3.88 (m, 3H), 3.93 (m, 1H), 3.99 (m, 1H), 4.05 (m, 1H), 4.11 (dd, *J* = 2.4, 12.3 Hz, 1H), 4.27 (dd, *J* = 5.1, 12.3 Hz, 1H), 4.80 (d, *J* = 1.7 Hz, 1H), 4.84 (d, *J* = 1.7 Hz, 1H), 4.86 (d, *J* = 1.4 Hz, 1H), 5.21 – 5.98 (m, 9H); ^{13}C{^{1}H} NMR ($CDCl_3$, 100 MHz) δ 20.6, 20.7 (3C), 20.8 (5C), 20.9, 28.7 (t), 48.2 (t), 62.3 (t), 64.9 (t), 65.9, 66.3, 66.4, 66.5 (2C, t), 68.6, 69.0 (2C), 69.2, 69.3 (2C), 69.4 (2C) 69.5, 97.4 (2C), 97.7, 169.6 (s), 169.7 (s), 169.8 (3C, s), 169.9 (2C, s), 170.2 (s), 170.3 (s), 170.6 (s); HRMS (ESI) calculated for $C_{41}H_{57}N3O_{26}Na$ $[M+Na]^+$ 1030.3122, found 1020.3121 (Δ = -0.1 ppm).

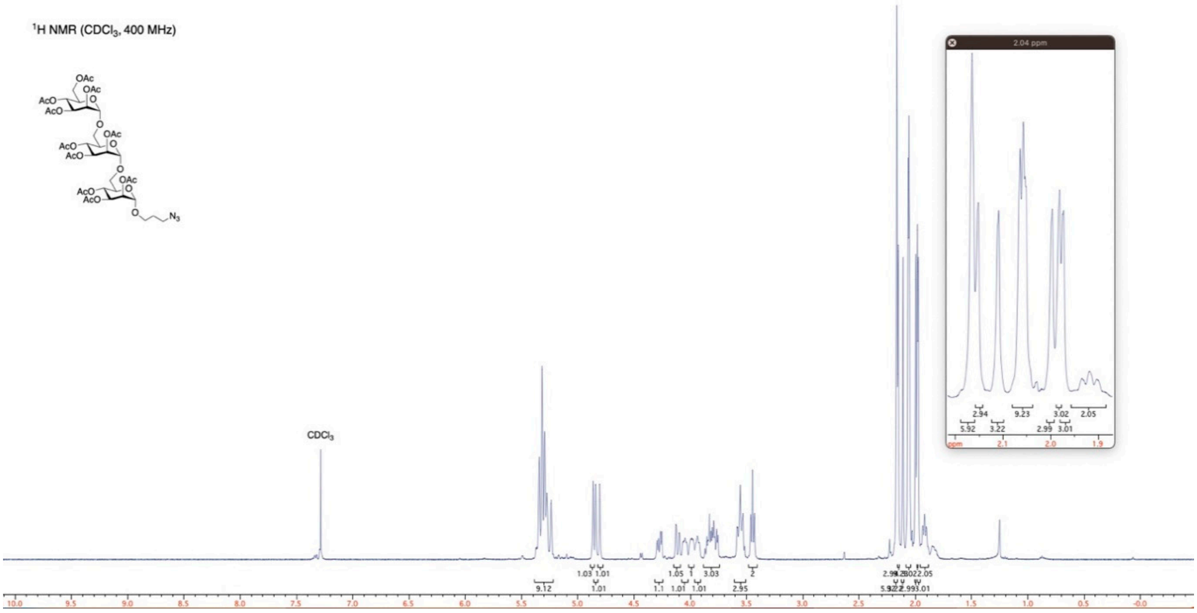

^{1}H NMR (CDCl$_3$, 400 MHz)
CDCl$_3$

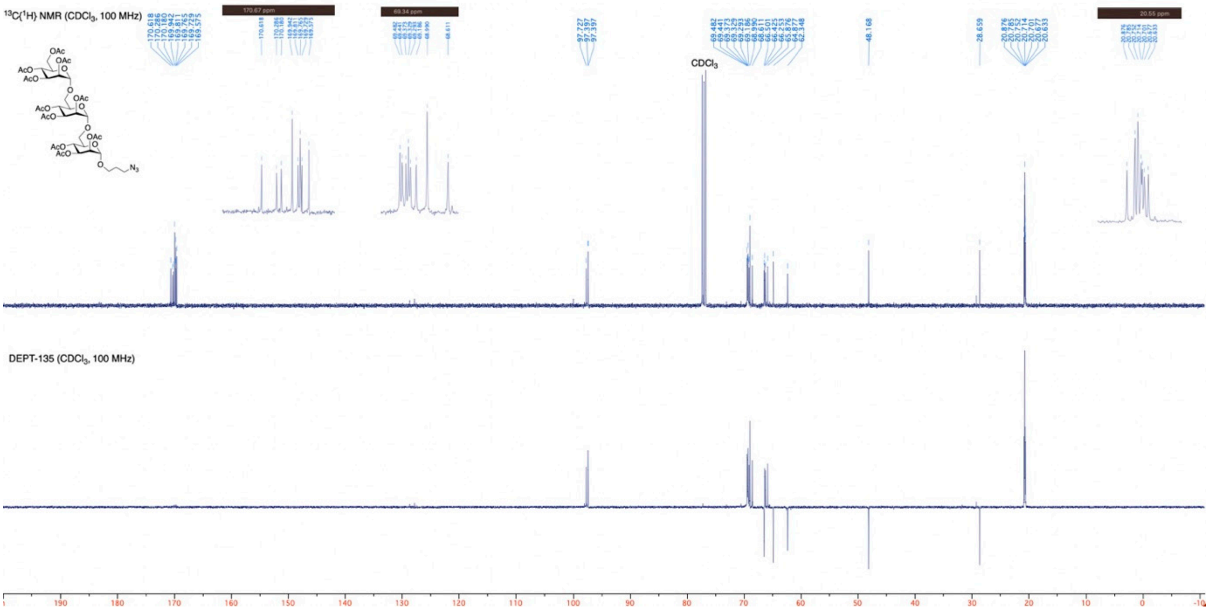

^{13}C{^{1}H} NMR (CDCl$_3$, 100 MHz)
CDCl$_3$
DEPT-135 (CDCl$_3$, 100 MHz)

3-Aminopropryl α-D-mannopyranosyl-(1→6)-α-D-mannopyranosyl-(1→6)-α-D-mannopyranoside (18)

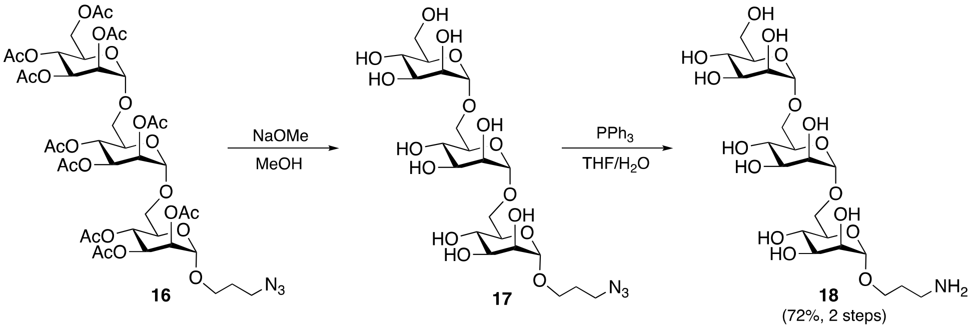

A solution of **16** (25mg, 0.025 mmol, 1 eq.) in MeOH (0.05M) was cooled at 0°C and added with NaOMe (6.7mg, 0.124 mmol, 5 eq.).

The reaction was stirred under nitrogen atmosphere until completion (checked by MS analysis), then acidic Dowex resin (Dowex 50W X8) was added in proportion until a pH of 6-7 was reached.

The resin was filtered off, washed with methanol, and the organic phase evaporated, leaving **17,** which was used in the next synthetic step without further purification.

In a round bottom flask, PPh_3 (12 mg, 0.045 mmol, 1.8eq.) was added to a solution of **17** in THF-H_2O (85:15, 0.05M) under stirring. The reaction was heated at 75°C and monitored by MS analysis.

The THF was then evaporated, the mixture diluted with 300 µL of water, and the solid precipitate filtered off. The filtrate was evaporated to dryness affording **18** (10.0mg, Y: 72%, over 2 passages).

^{1}H NMR (D_2O, 400 MHz) δ 1.82 – 1.90 (m, 2H), 2.90 – 3.03 (m, 2H), 3.51 – 3.76 (m, 13H), 3.77 – 3.91 (m, 7H), 4.76 (d, *J* = 1.6 Hz, 1H), 4.78 (d, *J* = 1.6 Hz, 1H), 4.81 (d, *J* = 1.6 Hz, 1H); ^{13}C{^{1}H} NMR (D_2O, 100 MHz) δ 27.3 (t), 37.5 (t), 60.9 (t), 65.1 (t), 65.4 (t), 65.5 (t), 66.4, 66.5, 66.7, 69.8, 69.9 (2C), 70.5, 70.6, 70.8 (3C), 72.7, 99.2, 99.4, 99.9; HRMS (ESI) calculated for $C_{21}H_{40}NO_{16}$ $[M+H]^+$ 562.2342, found 562.2337 (Δ = -0.8 ppm).

^{1}H NMR (D_2O, 400 MHz)

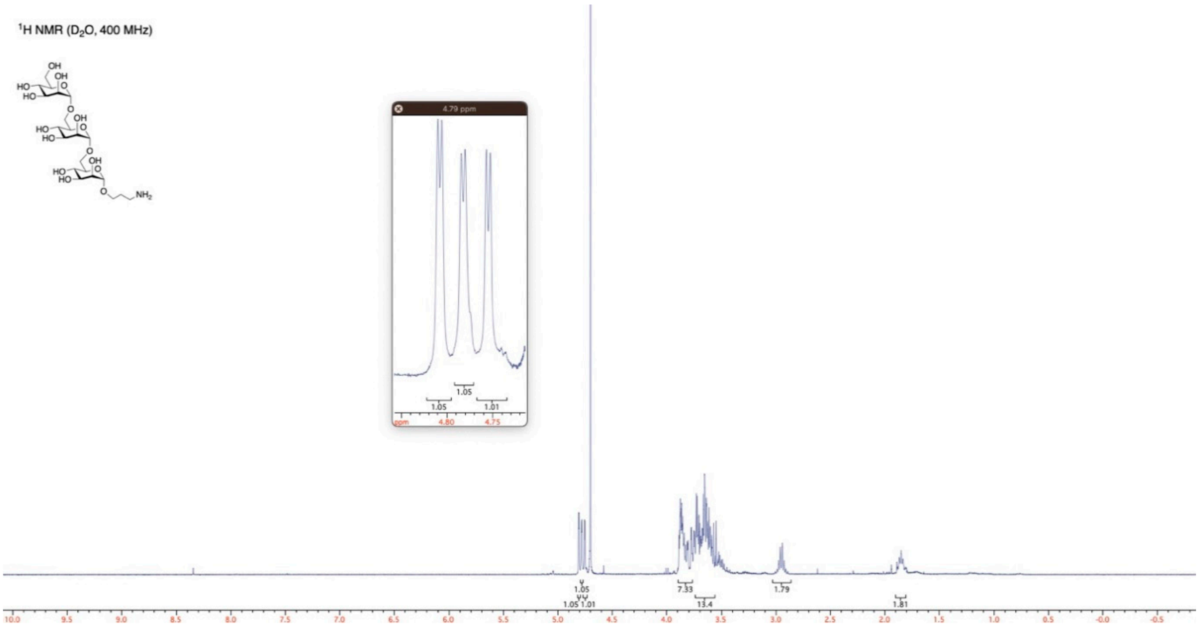

^{13}C{^{1}H} NMR (D_2O, 100 MHz)

DEPT-135 (D_2O, 100 MHz)

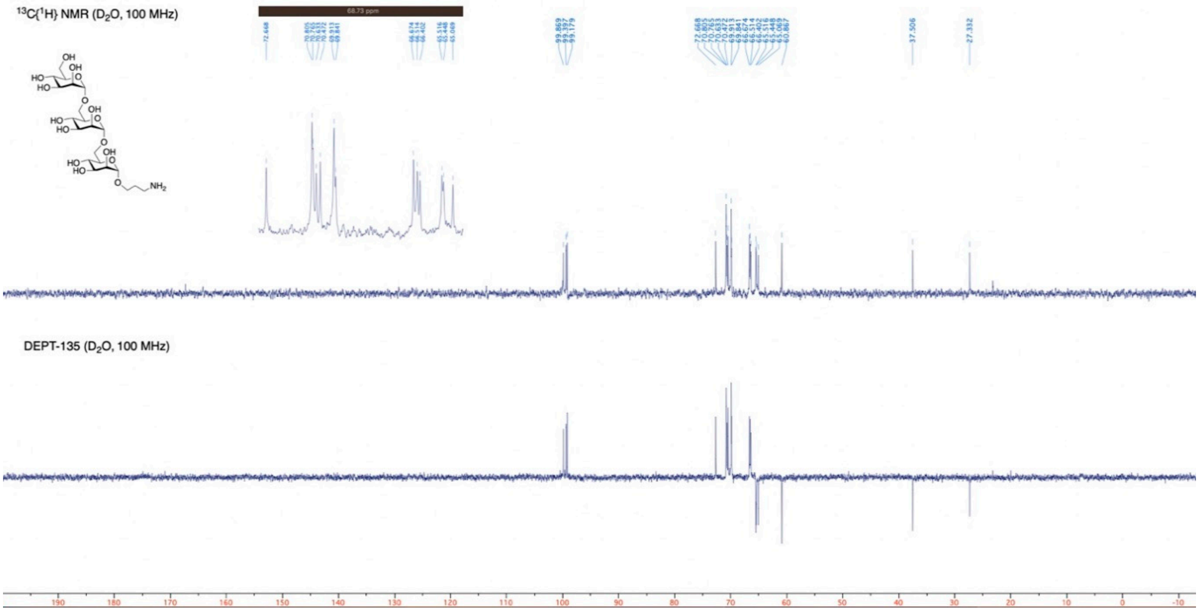

Supplement: Supplementary file 1 [file pharmaceutics-15-01321-s001.zip › pharmaceutics-2305776-supplementary.pdf]
